# Prediction of Maize Phenotypic Traits With Genomic and Environmental Predictors Using Gradient Boosting Frameworks

**DOI:** 10.3389/fpls.2021.699589

**Published:** 2021-11-11

**Authors:** Cathy C. Westhues, Gregory S. Mahone, Sofia da Silva, Patrick Thorwarth, Malthe Schmidt, Jan-Christoph Richter, Henner Simianer, Timothy M. Beissinger

**Affiliations:** ^1^Division of Plant Breeding Methodology, Department of Crop Sciences, University of Goettingen, Goettingen, Germany; ^2^Center for Integrated Breeding Research, University of Goettingen, Goettingen, Germany; ^3^Kleinwanzlebener Saatzucht (KWS) SAAT SE, Einbeck, Germany; ^4^Animal Breeding and Genetics Group, Department of Animal Sciences, University of Goettingen, Goettingen, Germany

**Keywords:** machine learning, genotype-by-environment interactions, gradient boosting, maize, yield, genomic prediction, plant breeding

## Abstract

The development of crop varieties with stable performance in future environmental conditions represents a critical challenge in the context of climate change. Environmental data collected at the field level, such as soil and climatic information, can be relevant to improve predictive ability in genomic prediction models by describing more precisely genotype-by-environment interactions, which represent a key component of the phenotypic response for complex crop agronomic traits. Modern predictive modeling approaches can efficiently handle various data types and are able to capture complex nonlinear relationships in large datasets. In particular, machine learning techniques have gained substantial interest in recent years. Here we examined the predictive ability of machine learning-based models for two phenotypic traits in maize using data collected by the Maize Genomes to Fields (G2F) Initiative. The data we analyzed consisted of multi-environment trials (METs) dispersed across the United States and Canada from 2014 to 2017. An assortment of soil- and weather-related variables was derived and used in prediction models alongside genotypic data. Linear random effects models were compared to a linear regularized regression method (*elastic net*) and to two nonlinear gradient boosting methods based on decision tree algorithms (*XGBoost, LightGBM*). These models were evaluated under four prediction problems: (1) tested and new genotypes in a new year; (2) only unobserved genotypes in a new year; (3) tested and new genotypes in a new site; (4) only unobserved genotypes in a new site. Accuracy in forecasting grain yield performance of new genotypes in a new year was improved by up to 20% over the baseline model by including environmental predictors with gradient boosting methods. For plant height, an enhancement of predictive ability could neither be observed by using machine learning-based methods nor by using detailed environmental information. An investigation of key environmental factors using gradient boosting frameworks also revealed that temperature at flowering stage, frequency and amount of water received during the vegetative and grain filling stage, and soil organic matter content appeared as important predictors for grain yield in our panel of environments.

## 1. Introduction

The development of environmental sensing technologies, including local weather stations, soil and crop sensors has progressively enabled field-level climate data to be incorporated into the analysis of plant breeding experiments (Tardieu et al., 2017; Ersoz et al., 2020; Crossa et al., 2021). When used to enhance genomic prediction, climate data can be useful to estimate the differential response of genotypes to new environmental conditions, i.e., genotype-by-environment interactions (GxE), almost omnipresent in multi-environment trial (MET) experiments (Cooper and DeLacy, 1994; Chenu, 2015). In plant breeding, an environment generally refers to the set of growing conditions associated with a given location in a given year. Various statistical models, such as factorial regression methods, have been developed to model genotype sensitivity to continuous environmental covariates (ECs) (van Eeuwijk et al., 1996; Malosetti et al., 2004) or even to simple geographic coordinates (Costa-Neto et al., 2020b) capturing primarily genotype-by-location interaction effects explained by crop management or soil characteristics.

Before the emergence of environmental data in breeding, large whole-genome marker datasets, generated by high-throughput genotyping platforms, have progressively enabled the routine implementation of genomic prediction (GP) methods (Haley and Visscher, 1998; Meuwissen et al., 2001). GP allows to predict performance of untested genotypes based on their genetic similarity, estimated with marker data, with other phenotyped genotypes. GP has since been expanded to achieve predictions in a multi-environment context, for instance by implementing a multivariate GBLUP approach (Burgueño et al., 2012) to use genetic correlations among environments. Despite the overall success of genomic prediction, a lingering challenge has regularly been to incorporate interactions between high-dimensional genomic data and high-dimensional environmental data. A solution proposed by Jarquín et al. (2014) is to use reaction norm models, where markers and environmental effects are modeled using covariance structures. Interactions between markers and environmental covariates are computed with the Hadamard product which avoids the need to fit all first-order interaction terms. This extension of the GBLUP GxE mixed effects models has been applied on a large number of datasets in different species (Pérez-Rodríguez et al., 2015, 2017; Jarquín et al., 2017; Sukumaran et al., 2017, 2018; Monteverde et al., 2019; Rincent et al., 2019; De Los Campos et al., 2020). Several studies have also focused on the integration of crop growth models in genomic prediction to better model the differential impact of abiotic stress depending on the crop developmental stage (Heslot et al., 2014a; Rincent et al., 2017, 2019). Rincent et al. (2019) proposed a method to select the optimal subset of ECs from the output of a crop growth model on the basis of the correlation between the environmental covariance matrix, which is based on ECs, and the covariance matrix between GxE interactivity of environments obtained by AMMI decomposition. Overall, many studies have found that using quantitative environmental information in genomic prediction models in the form of additional covariates can result in an enhancement of prediction accuracies (Heslot et al., 2014b; Jarquín et al., 2014; Malosetti et al., 2016; Millet et al., 2019; Monteverde et al., 2019; Costa-Neto et al., 2020a) and a better characterization of the genotype-by-environment interaction effects (Rogers et al., 2021).

However, modeling interaction effects with nonlinear techniques is a crucial topic that has not been conclusively explored for genomic prediction in MET. In particular, machine learning techniques have gained attention over the last two decades due to their ability to handle nonlinear effects (Hastie et al., 2009) and to uncover higher-order interactions between predictor variables (Lampa et al., 2014; Behravan et al., 2018). With machine learning algorithms, the mapping function linking input variables to the outcome—i.e., a phenotypic trait—is learned from training data and no strong assumptions about its form need to be explicitly formulated beforehand. Hence, these methods represent relatively flexible frameworks for data-driven integration of different data types. Among these new techniques, ensembles of trees, such as methods based on bagging (e.g., random forests), or on boosting (e.g., gradient boosted trees) have become increasingly popular. Ensemble methods designate predictive modeling techniques which aggregate the predictions of a group of base learners, and thereby generally allow better predictions than by using only the single best learner (Friedman, 2001; Hastie et al., 2009; Géron, 2019). Broad applications of these approaches include human disease prediction (Fukuda et al., 2013; Romagnoni et al., 2019; Yu et al., 2019; Kopitar et al., 2020), bioinformatics (Yu et al., 2019), ecology (Moisen et al., 2006; Elith et al., 2008) and agricultural forecasting (Fukuda et al., 2013; Delerce et al., 2016; Jeong et al., 2016; Crane-Droesch, 2018; Shahhosseini et al., 2020). In the field of genomic prediction, ensemble methods have progressively been used, as they appear especially interesting for capturing non-additive effects such as epistasis or dominance effects, which can be important for predicting complex phenotypic traits (Ogutu et al., 2011; González-Recio et al., 2013; Azodi et al., 2019; Abdollahi-Arpanahi et al., 2020). Abdollahi-Arpanahi et al. (2020) concluded from results obtained on both a real animal and simulated datasets that gradient boosting was the best predictive modeling approach when the genetic architecture included non-additive effects. While these new predictive modeling approaches can also potentially enable superior prediction results, special attention must be paid to an appropriate optimization of hyperparameters during the training phase in order to prevent overfitting on new test data (Friedman, 2001; Hastie et al., 2009; Géron, 2019).

In addition, these new predictive modeling frameworks, coupled with large volumes of environmental data, can provide powerful data mining opportunities to identify critical environmental factors affecting economically important phenotypic traits in the field. Much research has already been done to examine the expected impact of climate change on the vulnerability of major staple food crops. Extreme weather events are expected to happen at a higher frequency in the future, characterized for instance by heat waves or prolonged drought periods according to various climate scenarios (Rahmstorf et al., 2012; Trnka et al., 2014). When occurring at crucial crop developmental stages, risks for important yield losses are augmented. Different studies on maize have for instance reported a physiological sensitivity to higher temperatures, heightened during the reproductive phase, which often results in grain yield reduction when a certain threshold is exceeded (Cicchino et al., 2010; Butler and Huybers, 2015; Lizaso et al., 2018). In addition, nonlinear effects of environmental covariates, especially of temperature and precipitation on maize plants, have also been regularly described in the literature (Schlenker and Roberts, 2009; Mushore et al., 2017). Therefore, machine learning techniques break new ground to get an extended comprehension of the effect—both in direction and magnitude—of environmental conditions in the context of breeding for abiotic stress resilience.

Motivated by previous studies emphasizing the benefit of nonlinear methods, we tested two machine learning ensemble methods, based on gradient boosted trees, which, to our knowledge, have never been examined for data-driven predictions and interpretation using MET experimental datasets from the Maize Genomes to Fields initiative. The Maize Genomes to Fields (G2F) initiative (www.genomes2fields.org) includes yearly evaluations of inbred and hybrid maize across a large range of climatically-distinct regions in North America. The project makes publicly available phenotypic and genotypic (genotyping-by-sequencing datasets relating to the inbred lines) information, as well as weather (field weather stations), agronomic practices and soil data (Falcon et al., 2020; McFarland et al., 2020). The large number of phenotypic observations, and the assortment of various data types makes the application of machine learning models here particularly relevant to evaluate their performance, as well as their usefulness to disentangle hidden relationships. Our objectives in this study were (1) to evaluate recent gradient boosting methods for prediction of two phenotypic traits (plant height and grain yield) across four different cross-validations, and compare them to traditional prediction models classically used for multi-environment trials; (2) to examine if the use of environmental information, in addition to genomic predictor variables, could lead to a gain of predictive ability of genotype performance based on these various prediction models; and (3) to better understand the influence of some environmental factors on maize grain yield using tools derived from the machine learning framework.

## 2. Materials and Methods

### 2.1. Phenotypic Data Cleaning and Analysis

Phenotypic datasets (years 2014–2017) were downloaded from the official website of the Genomes to Fields project. The full dataset represents a large collection of trials located on the North-American continent run by different principal investigators and institutions, but the experimental design used for most of the hybrid trials was a randomized complete block design with two replications per environment. A total number of 71 trial experiments remained for further analysis (Supplementary Figure 1; Supplementary Table 1) after having eliminated environments with critical missing information, such as flowering time (Supplementary Table 2). Plots with low phenotypic quality, as interpreted by the researcher groups who collected field data, were removed before within-experiment analysis. Replicates within a same ID experiment but planted seven or more days apart were considered as different environments and treated as unreplicated environments, due to the difference in the weather conditions they experienced at their respective phenological stages.

Each environment (Year-Site combination) was independently analyzed to obtain best linear unbiased estimates (BLUEs) for each hybrid in each environment for grain yield, plant height and silking date. We performed this analysis with the *lme4* package (Bates et al., 2015) in R version 3.6.0 (R Core Team, 2019) based on the following model:


$$Y_{ij}=\mu +G_{i}+R_{j}+\varepsilon_{ij},$$

where *Y*_*ij*_ is the observed phenotypic response variable of the *i*-th hybrid genotype (G) in the *j*-th replicate (R), μ is the general mean, *G*_*i*_ is the effect of the *i*-th hybrid genotype, *R*_*j*_ is the effect of the *j*-th replicate and ε_*ij*_ is the error associated with the observation *Y*_*ij*_. We treated genotype as a fixed effect and replicate as a random effect.

Phenotypic observations with absolute studentized conditional residuals greater than three were identified as potential outliers and removed from the dataset. The plant material and phenotypic datasets are described in more details in previous publications (AlKhalifah et al., 2018; McFarland et al., 2020) and on the project website (https://www.genomes2fields.org/home/). Ultimately, 18,325 and 16,951 phenotypic observations for grain yield and plant height, respectively, with available silking date, genotypic and environmental data, were used as target response variable in the prediction models.

### 2.2. Genotypic Data

Genotype-by-sequencing (GBS) data of inbred lines used in Genomes to Fields hybrid experiments were downloaded on CyVerse. SNPs with more than two observed alleles were removed before analysis. Taxa with less than 70% site coverage and more than 8% heterozygosity were discarded. Monomorphic markers were removed, as were those missing or heterozygous in more than 5% of the parental lines. These filtering analyses were performed with TASSEL 5 (Bradbury et al., 2007). After filtering, 246,818 SNPs remained for analysis. These were imputed using the software LinkImpute (Money et al., 2015). The genotype matrix was coded as the number of minor alleles at each locus (0, 1, or 2). Markers with minor allele frequency less than 2% and in high linkage Disequilibrium (LD) were further removed using the pruning function of Plink (Purcell et al., 2007) with a window of size 100 markers, a step of 5, and a LD threshold of 0.99. *In silico* genotypes of maize hybrids, for which phenotypic data had been analyzed, were constructed based on the processed genotypes of parental lines, and a final minor allele frequency filtering of 2% was applied. The final hybrid genotype dataset contained 107,399 SNPs characterizing 2,033 hybrids. Additional details regarding the genotype-by-sequencing procedure implemented by the Genomes to Fields project has been previously published (Gage et al., 2017).

### 2.3. Weather Data

All field experiment locations in the Genomes to Fields project had a WatchdogTM Model 2700 weather station (Spectrum Technologies Inc., East-Plainfield, Illinois, 60585, USA) on-site. Weather records were recorded every 30 min during the growing season. Measurements for air temperature (°C), relative humidity (%), rainfall (mm), solar radiation (W/m2) and wind speed (m/s) were specifically analyzed. In-field weather station measurements provide climatic information of a very localized scale in comparison to weather service stations. Therefore, we prioritized the use of weather-station data whenever data quality criteria were fulfilled and the proportion of missing data was reasonable. When quality criteria were not met, weather data was acquired from nearby weather service stations.

In the first step, we summarized the hourly or semi-hourly records for each climatic variable on a daily basis using various quality control criteria (consistent number of weather records per day; threshold tests; persistence tests, i.e., flagging observations with null variability during the day; internal consistency tests, i.e., verification of the relation between measured variables). These criteria were applied based on the recommendations from the official published guidelines on quality control procedures for data acquired from weather stations (Zahumenský, 2004; Estévez et al., 2011) and are detailed in Supplementary Table 3. Data from the field weather station were compared against weather data obtained from public climate summaries to check for possible large data divergences and to fill out missing values. Data from the Global Historical Climatology Network (GHCN) and from the Global Surface Summary of the Day (GSOD) were retrieved from the National Oceanic and Atmospheric Administration (NOAA) website to investigate American locations, while data for Canadian locations were downloaded from the Environment and Climate Change Canada (ECCC) website, based each time on a 70-kilometer radius from the geographic coordinates for each field experiment. In case data from the field weather station data were missing or assigned as erroneous, data from the closest publicly accessible weather station were used, if it was located less than 2 km from the field. If the distance to the nearest station was large, interpolation by spatio-temporal kriging or inverse distance weighting was performed using the R package *gstat* to impute the missing data (Pebesma, 2004; Gräler et al., 2016). For wind data, we only used results obtained from inverse distance weighting because of the consistency regarding the standard height measurement obtained from GSOD data. Similarly, in-field weather stations solar radiation data were characterized by a high percentage of missing values and inconsistencies; we used instead the R package nasapower (Sparks, 2018), which enables an easy access to NASA POWER surface solar radiation energy data. Some environments were irrigated: for those of which the precise amount was tracked during the growing season, these data were added to the final daily precipitation data.

Hence, the daily weather data consisted of the daily maximum, minimum and mean temperature (average of minimum and maximum daily temperatures), average wind speed, precipitation, humidity, incoming solar radiation. Based on these processed weather data, we were then able to calculate the daily growing degrees (Baskerville and Emin, 1969), the photothermal time (product between GDs and day length in hours, for each day, also referred as an environmental index; Li et al., 2018), the mean vapor pressure deficit, the reference evapotranspiration (*ET*_0_) using FAO-56 Penman-Monteith method (Allen et al., 1998). These latter variables were computed because they incorporate crop physiological parameters which make them sometimes more relevant than the initial weather data.

### 2.4. Derivation of Environmental Variables per Hybrid Growth Stage

The next step was to obtain pertinent environmental predictors from daily weather summaries for the predictive modeling framework. The objective was to relate each hybrid phenotypic performance (e.g., yield) in a particular environment, individually characterized by its specific flowering dates, to the corresponding weather series during the growing season. To develop a unified framework across the different growing season lengths, which varied throughout locations and years, we used three critical maize growth stages, as was performed in previous similar work for other crops (Heslot et al., 2014b; Delerce et al., 2016; Gillberg et al., 2019; Monteverde et al., 2019). This approach was needed to account for the differential impact of weather-based variables according to the crop developmental stage. Each intermediate plant developmental stage could not be precisely determined since visual scoring for all stages is in practice highly time-consuming and expensive. However, the sowing date and the flowering date, i.e., when 50% of plants in a plot have visible silk, were recorded for each hybrid kept after phenotypic data analysis. Based on these known dates, three hybrid maize growth periods could be estimated: vegetative (from the planting date to 1 week before the 50% silking date); flowering (from 1 week before 50% silking date to 2 weeks after that date, which corresponds approximately to the end of the pollination period); and the grain filling stage (from the end of the flowering period to 65 days after, after which maturity should be reached). By definition, these three periods do not overlap. The typical duration of the grain filling stage varies according to the hybrid and the environment; nonetheless, based on literature and agronomic knowledge, the corn plant is normally at physiological maturity (R6) about 55–65 days after silking (Ritchie et al., 1993).

Based on these dates, 13 weather-based environmental predictor variables were computed for each phenological stage and therefore were both environment- and hybrid-specific (Table 1). We included stress covariates related to heat, as it is expected that an excess of heat can be detrimental, especially during the flowering stage, and results in a lower yield. To examine the presence of clusters of environments based on climatic similarity, a principal component analysis on the weather-based covariates using the R package factoextra (Kassambara and Mundt, 2017) was applied.

**Table 1:** Environmental predictor variables used in the prediction models.

**Acronym**	**General description**
P.V, P.F, P.G	Accumulated precipitation + irrigation (mm) by growth period
FreqP5.V, FreqP5.F, FreqP5.G	Frequency of days with more than 5 mm precipitation by growth period
MeanT.V, MeanT.F, MeanT.G	Average of daily mean temperature (°C) by growth period
MinT.V, MinT.F, MinT.G	Average of minimum daily temperature (°C) by growth period
MaxT.V, MaxT.F, MaxT.G	Average of maximum daily temperature (°C) by growth period
GDD.V, GDD.F, GDD.G	Cumulative growing degree days, Base 10°C (°C) by growth period
Photothermal.Time.V, Photothermal.Time.F, Photothermal.Time.G	Cumulative photothermal time (GDD x Day Length) by growth period
FreqMaxT30.V, FreqMaxT30.F, FreqMaxT30.G	Frequency of days with maximum temperature above 30°C by growth period
FreqMaxT35.V, FreqMaxT35.F, FreqMaxT35.G	Frequency of days with maximum temperature above 35°C by growth period
St30.V, St30.F, St30.G	Sum of the daily maximal temperatures above 30°C (°C)
CumSumET0.V, CumSumET0.F, CumSumET0.G	Accumulated reference evapotranspiration (mm), under standard conditions, according to the FA0-56 Penman-Monteith methodology for each growth period
CumDailyWaterBalance.V, CumDailyWaterBalance.F, CumDailyWaterBalance.G	Cumulative daily water balance, i.e., daily precipitation + irrigation - daily reference evapotranspiration (mm)
Sdrad.V, Sdrad.F, Sdrad.G	Accumulated incoming daily solar radiation (MJ m-2 day-1) by growth period
SandProp.SC	Sand composition (%)
Silt.Prop.SC	Silt composition (%)
ClayProp.SC	Clay composition (%)
OM.SC	Percentage of organic matter (%)

*The suffixes refer to: V, vegetative period; F, flowering period; G, grain fill period; SC, soil covariate*.

In addition to climatic variables, our framework accommodates four soil-based environmental variables: soil quality types (percentages of sand, silt, and clay composition) and percentage of soil organic matter. The majority of the soil information originates from the soil samples realized at each G2F field location; otherwise, when the location presented missing information, we defined an area of interest based on field geographical coordinates using the Web Soil Survey application for American locations, and the web mapping application Agricultural Information Atlas for Canadian locations, and retrieved the aforementioned data of interest. In the rest of the paper, the abbreviation “W” refers to the set of weather-based and soil-based environmental covariates. For the trait plant height, weather-based covariates from the grain filling stage were not used as explanatory variable for prediction, since this trait was usually measured shortly after flowering time.

### 2.5. Prediction Models Implemented

#### 2.5.1. Linear Random Effects Models (LRE Models)

In multi-environment trial analysis and plant breeding experiments, linear random effects models, abbreviated to LRE models thereafter, are often used as genomic prediction models and were compared in this study with machine learning techniques, according to the models outlined in Jarquín et al. (2014). In particular, GxE can be modeled with a covariance function equal to the product of two random linear functions of markers and of environmental covariates, which is equivalent to a reaction norm model (Jarquín et al., 2014). An environment always refers to a Site x Year combination.

**Main effects models**

*(1) Model G + E: Marker + Environment Main Effects (baseline model)*

The response variable is modeled as the sum of an overall mean (μ), plus random deviations due to the environment *E*_*i*_ and to the genotypic random effect of the *j*th hybrid genotype *g*_*j*_ based on marker covariates (G-BLUP component), plus an error term ε_*ij*_:


(1) $$\begin{array}{r} y_{ij}=\mu +E_{i}+g_{j}+\varepsilon_{ij}, \end{array}$$

where $E_{i}\overset{IID}{\sim }N\left(0,\sigma_{E}^{2}\right)$, $\text{g}\overset{IID}{\sim }N\left(0,\text{G}\sigma_{g}^{2}\right)$ and $\varepsilon_{ij}\overset{IID}{\sim }N\left(0,\sigma_{\varepsilon }^{2}\right)$, and N(.,.) denotes a normally distributed random variable, IID stands for independent and identically distributed, and $\sigma_{E}^{2}$, $\sigma_{g}^{2}$ are the corresponding environmental and genomic variances, respectively.

*g*_*j*_ corresponds to a regression on marker covariates of the form $g_{j}=\overset{p}{\underset{m=1}{\sum }}x_{jm}b_{m}$, linear combination of *p* markers and their respective marker effects. Marker effects were regarded as IID draws from normal distributions of the form $b_{m}\overset{IID}{\sim }N\left(0,\sigma_{b}^{2}\right)$, *m = 1,...,p*. The vector **g = Xb** follows a multivariate normal density with null mean and covariance-matrix $Cov\left(\text{g}\right)=\text{G}\sigma_{g}^{2}$, where $G=\frac{XX^{\prime }}{p}$ is the genomic relationship matrix, X representing the centered and standardized genotype matrix and *p* is the total number of markers.

*(2) Model G + S: Marker + Site Main Effects*

The present model allows to gain information from a site evaluated over several years, as it includes the site effect:


(2) $$\begin{array}{r} y_{kj}=\mu +S_{k}+g_{j}+\varepsilon_{kj} \end{array}$$

Here *y*_*kj*_ corresponds to the phenotypic response of the *j*th genotype in the *k*th site with $S_{k}\overset{IID}{\sim }N\left(0,\sigma_{S}^{2}\right)$, *k = 1,...,K*.

*(3) Model G+E+W: Marker + Ennvironment + Environmental Covariates Main Effects*

This model incorporates additionally the main effect of the environmental covariates (including the longitude and latitude coordinates). We can model the environmental effects by a random regression on the ECs (**W**), that represents the environmental conditions experienced by each hybrid in each environment: $w_{ij}=\overset{Q}{\underset{q=1}{\sum }}W_{ijq}\gamma_{q}$, where *W*_*ijq*_ is the value of the *q*th EC evaluated in the *ij*th environment x hybrid combination, γ_*q*_ is the main effect of the corresponding EC, and Q is the total number of ECs. We considered the effects of the ECs as IID draws from normal densities, i.e., $\gamma_{q}\sim N\left(0,\sigma_{\gamma }^{2}\right)$. Consequently, the vector **w** = **W*γ*** follows a multivariate normal distribution with null mean and covariance matrix $\Omega \sigma_{w}^{2}$, where **Ω** ∝ **WW′**, and the matrix **W**, which is centered and standardized, contains the values of the ECs. The model becomes then:


(3) $$\begin{array}{r} y_{ij}=\mu +E_{i}+g_{j}+w_{ij}+\varepsilon_{ij} \end{array}$$

with $\text{w}\sim N\left(\text{0},\Omega \sigma_{w}^{2}\right)$.

In this model, as explained in Jarquín et al. (2014), environmental effects are subdivided in two components, one that originates from the regression on numeric environmental variables, and one due to deviations from the Year-Site combination effect which cannot be accounted for by the ECs. Indeed, the environmental variables might not be able to fully explain the differences across environments. The modeling of the covariance matrices **Ω** and **G** allows to borrow information between environments and between hybrid genotypes, respectively.

**Models with interaction**

*(4) Model G+E+GxE: main effects G+E with Genomic x Environment Interaction*

The model G+E was extended by including the interaction term between environments and markers (GxE):


(4) $$\begin{array}{r} y_{ij}=\mu +E_{i}+g_{j}+gE_{ij}+\varepsilon_{ij} \end{array}$$

with $gE\;\sim N(0,[Z_{g}GZ_{g}^{\prime }]{}^{\circ}[Z_{E}Z_{E}^{\prime }]\sigma_{gE}^{2}),\varepsilon_{ij}\overset{IID}{\sim }N(0,\sigma_{\varepsilon }^{2})$, where ***Z*_*g*_** and ***Z*_*E*_** are the design matrices that connect the phenotype entries with hybrid genotypes and with environments, respectively; $\sigma_{gE}^{2}$ is the variance component of the *gE*_*ij*_ interaction term; and ° denotes the Hadamard product between two matrices.

*(5) Model G+S+GxS: main effects G+S with Genomic x Site Interaction*

Similar to the previous model, this model extends model G+S by including the interaction term between sites and markers (GxS):


(5) $$\begin{array}{r} y_{kj}=\mu +S_{k}+g_{j}+gS_{kj}+\varepsilon_{kj} \end{array}$$

where $gS\;\sim N(0,[Z_{g}GZ_{g}^{\prime }]{}^{\circ}[Z_{S}Z_{S}^{\prime }]\sigma_{gS}^{2}),\varepsilon_{kj}\overset{IID}{\sim }N(0,\sigma_{\varepsilon }^{2})$, where ***Z*_*S*_** and $\sigma_{gS}^{2}$ are the design matrix for sites and the associated variance component for this interaction, respectively.

*(6) Model G+E+S+Y+GxS+GxY+GxE: main effects G+E+S+Y with Genomic x Environment Interaction, Genomic x Site Interaction and Genomic x Year Interaction*

This model corresponds to the most complete model using only basic GxE information (year and site information) about environments:


(6) $$\begin{array}{r} y_{jkm}=\mu +g_{j}+S_{k}+Y_{m}+E_{km}+gS_{jk}+gY_{jm}+gE_{jkm}+\varepsilon_{jkm} \end{array}$$

where $gY\;\sim N(0,[Z_{g}GZ_{g}^{\prime }]{}^{\circ}[Z_{Y}Z_{Y}^{\prime }]\sigma_{gY}^{2}),\varepsilon_{kj}\overset{IID}{\sim }N(0,\sigma_{\varepsilon }^{2})$, where ***Z*_*Y*_** and $\sigma_{gY}^{2}$ are the design matrix for years and the associated variance component for this interaction, respectively.

*(7) Model G+E+W+GxW: main effects G+E+W with interactions between markers and environmental covariates*

The model G+E+W was extended by adding the interaction between genomic markers and environmental covariates. Jarquín et al. (2014) demonstrated that this interaction term induced by the reaction-norm model can be described by a covariance structure which corresponds, under standard assumptions, to the Hadamard product of two covariance structures: one characterizing the relationships between lines based on markers information (e.g., **G**), and one describing the environmental resemblance based on ECs (e.g., ***Ω***). The vector of random effects, denoted **gw** represents the interaction terms between markers and ECs, is assumed to follow a multivariate normal distribution with null mean and covariance structure $[Z_{g}GZ_{g}^{\prime }]{}^{\circ}\Omega$. The model can be expressed as follows:


(7) $$\begin{array}{r} y_{ij}=\mu +E_{i}+g_{j}+w_{ij}+gw_{ij}+\varepsilon_{ij}, \end{array}$$

with $gw\sim N(0,[Z_{g}GZ_{g}^{\prime }]{}^{\circ}\Omega \sigma_{gw}^{2}$).

*(8) Model G+E+W+GxW+GxE: main effects G+E+W with Genomic x Environment Interaction and Genomic x Environmental Covariates Interaction*

The interaction term *gE*_*ij*_ is incorporated in this model, because some GxE might not be completely captured by the interaction term *gw*_*ij*_, and the model becomes:


(8) $$\begin{array}{r} y_{ij}=\mu +E_{i}+g_{j}+w_{ij}+gw_{ij}+gE_{ij}+\varepsilon_{ij} \end{array}$$

Main and interactions effects included in the different models described above are summarized in Supplementary Table 5. Models using W, i.e., the matrix of environmental covariates, were tested with and without longitude and latitude data included. Additional combinations of main effects and interactions not detailed here were also evaluated and results are presented as Supplementary Material. These models were implemented in a Bayesian framework using the R package BGLR (Pérez and de Los Campos, 2014), for which the MCMC algorithm was run for 42,000 iterations and the first 2000 cycles were removed as burn-in with thinning equal to 5.

#### 2.5.2. Machine Learning Based-Methods Used

The potential of machine learning models was explored using the following three algorithms: the linear regularized Elastic Net (Zou and Hastie, 2005), XGBoost (Chen and Guestrin, 2016) and LightGBM (Ke et al., 2017). All the machine learning regression models were conducted in R version 3.6.1 (R Core Team, 2019) using the tidymodels framework (Kuhn and Wickham, 2020) and wrapper functions of treesnip (https://github.com/curso-r/treesnip/). Elastic net is a regularized linear regression method that has proven to be useful with datasets characterized by multicollinearity to identify the most relevant predictor variables as well as reducing the computing time (Zou and Hastie, 2005). It corresponds to a linear combination of two penalty terms: the lasso (L1 regularization), noted $||\beta {||}_{1}=\overset{p}{\underset{j=1}{\sum }}|\beta_{j}|$ and the ridge (L2 regularization), noted $||\beta {||}_{2}^{2}=\overset{p}{\underset{j=1}{\sum }}\beta_{j}^{2}$. While the L2 penalty tends to contract the coefficients of highly correlated features toward each other, the L1 penalty supports a sparse solution, as many coefficients are zeroed. However, this method does not account for interactions between features.

Originally introduced by Friedman (2001), gradient boosting approach sequentially builds an ensemble of decision trees, with each new tree improving the predictions of the previous one by fitting on its residual errors. Two implementations of gradient boosting of decision trees (GBDT) for regression were used: Light Gradient Boosting Machine (LightGBM) and eXtreme Gradient Boosting (XGBoost). The two GBDT frameworks stand out from other similar boosting algorithms regarding their efficiency, which can be achieved by their common implementation of a histogram-based method for split finding, which groups continuous features into discrete bins. Hence, the algorithm does not iterate through all feature values, which is extremely time-consuming, but instead performs splitting on the bins. This speeds up training for very large datasets, as well as reducing memory usage. LightGBM, developed more recently, incorporates additional features, among others a downsampling during the training on basis of gradients. GBDT frameworks can handle well various types of data (binary, continuous data), and they are relatively robust to the effects of outliers among predictor variables (Hastie et al., 2009). Decision trees can capture, by construction, higher-order interactions between features, as well as nonlinear relationships between predictors and response variable (Friedman, 2001). Hence, interactions do not need to be explicitly provided as input data, since new splits are built conditional on preceding splits made on other predictors.

#### 2.5.3. Data Pre-processing for Machine Learning-Based Models

For data processing, we used the R package recipes (Kuhn and Wickham, 2020). To reduce genomic data dimensionality, we did not input SNP data into our prediction models directly. Instead, we used the top 275 or 350 principal components (PCs) of SNP data, for the traits grain yield and plant height, respectively. This set of PCs was chosen after evaluation of the predictive ability using different sets of top PCs explaining a various proportion of the variance in the data. Covariates which had no variance were removed using the step_nzv function. Retained covariates were standardized to zero mean and unit variance. As for linear random effect models, we tested the influence on prediction of longitude and latitude data by including and removing them as predictor variables across the different cross-validation scenarios. The year was also included as an input variable as a predictor variable in some models to account for environmental variation not fully captured by environmental covariates. In that case, the factor variable was converted into four new variables corresponding to each level of the original predictor. To model the site effect in models without numerical environmental information, we used the simple geographic coordinates of each location instead of using its label. Indeed, in decision trees, the use of a categorical predictor with a high number of levels can lead to overfitting (Hastie et al., 2009).

#### 2.5.4. Optimization of Hyperparameters and Hyperparameter Importance for Machine Learning-Based Models

**Figure 1:**
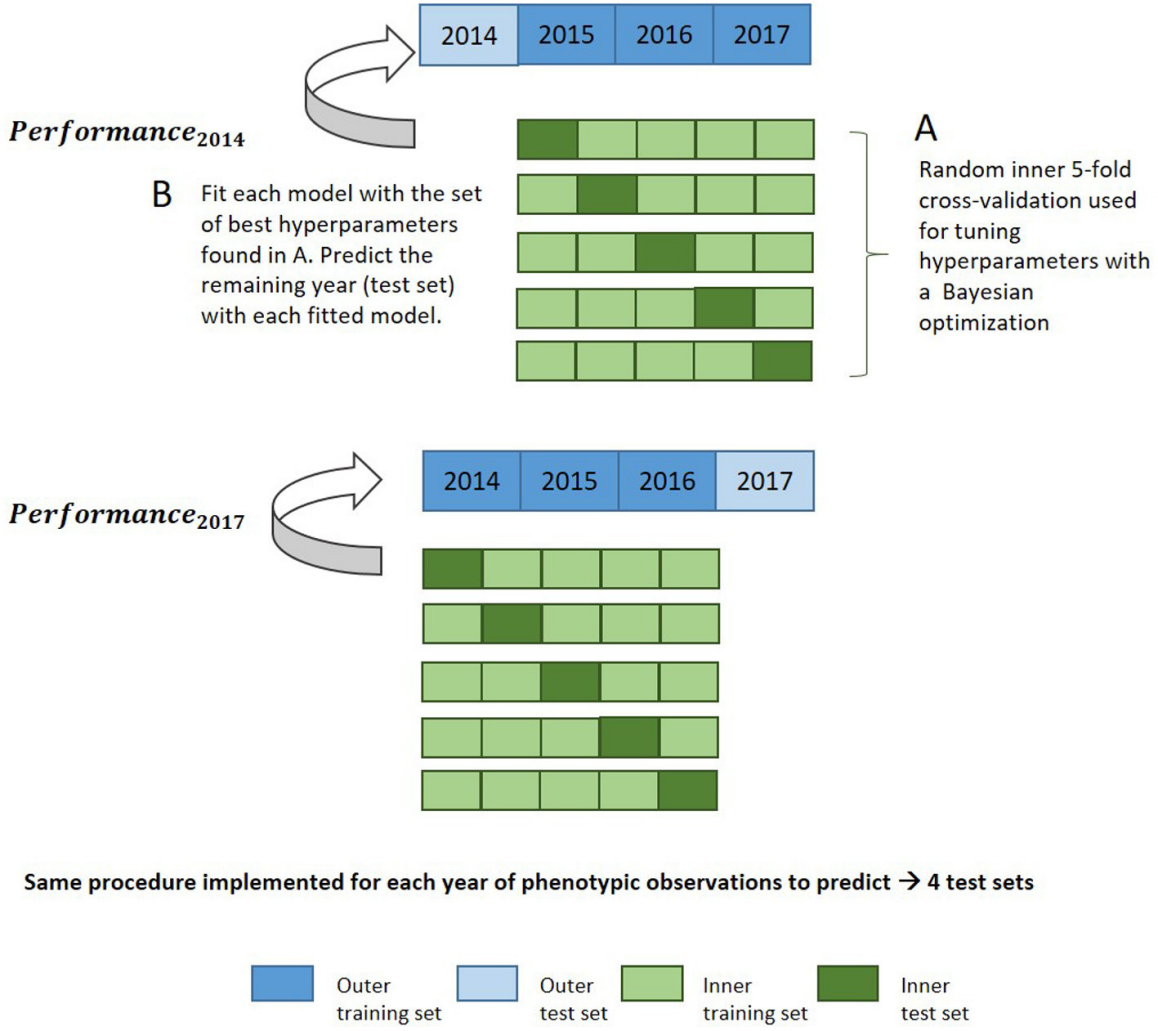
Nested cross-validation diagram for evaluation of model performance in the leave-1-year-out CV scheme with a machine learning approach.

Bayesian optimization using an iterative Gaussian process was used for hyperparameter tuning. It represents a much faster approach than grid search while allowing more flexibility in how the parameter space is covered. The Gaussian process builds a probability model based on an initial set of performance metrics obtained for various hyperparameter combinations during an initialization step, and predicts new tuning hyperparameters to test based on these previous results (Williams and Rasmussen, 2006; Snoek et al., 2012). Bayesian optimization incorporates prior assumptions on model parameter distribution and update it after each iteration, seeking to minimize the root mean square error (RMSE). Hyperparameter tuning was evaluated with 30 iterations under resampling based on a fivefold cross-validation (CV) with two repeats on the training set. Supplementary Table 4 indicates the set of hyperparameters tuned for each method during this optimization step. This set of hyperparameters was then used to fit the whole training data and predict the test set, which was unused during the optimization of hyperparameters. The general procedure for this nested cross-validation is illustrated in Figure 1. Fine-tuning of hyperparameters is required in order to prevent overfitting and to achieve the best prediction accuracy and representation of the data.

In addition, we examined the role of each hyperparameter on the overall model performance. This analysis provide insights into the most important hyperparameters to primarily tune in order to yield accurate models. We focus here on the LightGBM algorithm and XGBoost. A method based on random forests and functional ANOVA (fANOVA) was proposed by Hutter et al. (2014) to quantify the marginal contribution of each hyperparameter and pairwise interaction effects. Briefly, we used the output table of performance metrics of each algorithm with different hyperparameter combinations, which was obtained during the optimization step. The metric (root mean square error) is then used as target variable while hyperparameters represent the explaining variables to fit a random forest algorithm. fANOVA is then applied to evaluate the importance of each hyperparameter used in the grid search.

#### 2.5.5. Assessment of Prediction Accuracy for New Environments

In order to mimic real plant breeding problems, we considered four different cross-validation strategies aiming at predicting genotypes in environments that were never tested before, namely CV0-Year, CV0-Site, CV00-Year, and CV00-Site, described in Jarquín et al. (2017). The CV0 cross-validation scheme allows to borrow information in the training set about the performance of predicted genotypes in other tested environments, while the CV00 cross-validation scheme consists of the prediction of newly developed genotypes. This means that for implementation of the CV00 cross-validation, any observation from a genotype included in the test set (i.e., new environments) was removed from the training set. Predictions of untested genotypes can be achieved by exploiting information from marker data on genetic similarities between genotypes from the training set and from the test set. Four scenarios in total were examined, which differ according to whether site or year were used to build the test set, and to the degree of relationship between training and test set: (1) CV0-Year, where phenotypic information about the performance of genotypes evaluated in the same year was masked; (2) CV00-Year, where phenotypic information about the performance of any genotypes present in the test set in other years was additionally masked; (3) CV0-Site, where phenotypic information about the performance of genotypes evaluated in the same site was masked and (4) CV00-Year, where phenotypic information about the performance of any genotypes present in the test set in other sites was additionally masked. In this procedure, the number of observations contained in each outer fold is not the same, due to the unbalanced character of the dataset. This approach reflects a common issue arising in multi-environment plant breeding trials, as all selection candidates cannot be grown in all environments. However, we can ensure a fair model comparison by having the same data splits across tested models.

Regarding evaluation metrics, we define the prediction accuracy as the Pearson correlation between the predicted and the observed performance in a given environment, i.e., correlations were computed on a trial basis.

In order to take into account the difference in sample sizes between environments, we evaluated the weighted average predictive ability across environments according to Tiezzi et al. (2017), for each combination of prediction model, predictor variables and trait, as following:


$$r_{w}=\frac{\sum_{j=1}^{J}\frac{r_{j}}{V\left(r_{j}\right)}}{\sum_{j=1}^{J}\frac{1}{V\left(r_{j}\right)}},$$

with *r*_*j*_ the Pearson's correlation between predicted and observed values at the *j*^*th*^ environment, *V*(*r*_*j*_)$=\frac{1-r_{j}^{2}}{n_{j}-2}$ its sampling variance and *n*_*j*_ the total number of phenotypic observations in the *j*^*th*^ environment.

### 2.6. Variable Importance and Partial Dependence Plots for Grain Yield

We used the gain metric to quantify the feature importance in the XGBoost model fitted to the full dataset. This metric corresponds to the relative contribution of the variable to the ensemble model, calculated by considering each variable's contribution for each boosting iteration. A superior value of the gain for one feature compared to another feature means that this feature is more important to generate a‘prediction.

Overall partial dependence plots (PDPs) were computed using the R package DALEX (Biecek, 2018) using the four trained datasets from the CV0-Year scheme and the full dataset. PDPs are relevant to study how the predicted outcome of a machine learning model is partially influenced by a subset of explanatory variables of interest, by marginalizing over the values of all other variables.

The partial dependence profile of *f(X)* is defined as following by Friedman (2001):


$$f_{S}\left(X_{S}\right)=E_{X_{C}}f\left(X_{S},X_{C}\right),$$

where the *X*_*S*_ represents the set of input predictor variables for which the effect on the prediction is analyzed, and *X*_*C*_ represent the complement set of other predictor variables used in the model. The following partial function can be used as an estimator:


$$f_{S}\left(X_{S}\right)=\frac{1}{N}\overset{N}{\underset{i=1}{\sum }}f\left(X_{S},x_{iC}\right),$$

where *x*_1*C*_, *x*_2*C*_, ..., *x*_*NC*_ are the values of *X*_*C*_ observed in the training data. This means that we estimate this expected value as the average of the model predictions, over the joint distribution of variables in *X*_*C*_, when the set of joint values in *X*_*S*_ is fixed. As emphasized by Hastie et al. (2009), partial dependence functions represent hence the influence of *X*_*S*_ on *f(X)*, after taking into account the average effects of the other variables *X*_*C*_ on *f(X)*.

### 2.7. Code Availability

A Github repository containing the various R scripts and Bash scripts used for phenotypic analysis, processing of weather data, spatio-temporal interpolation of missing weather data, and predictive modeling is available: https://github.com/cjubin/G2F_data.

## 3. Results

### 3.1. Variability of Climatic Conditions in the Panel of Environments

**Figure 2:**
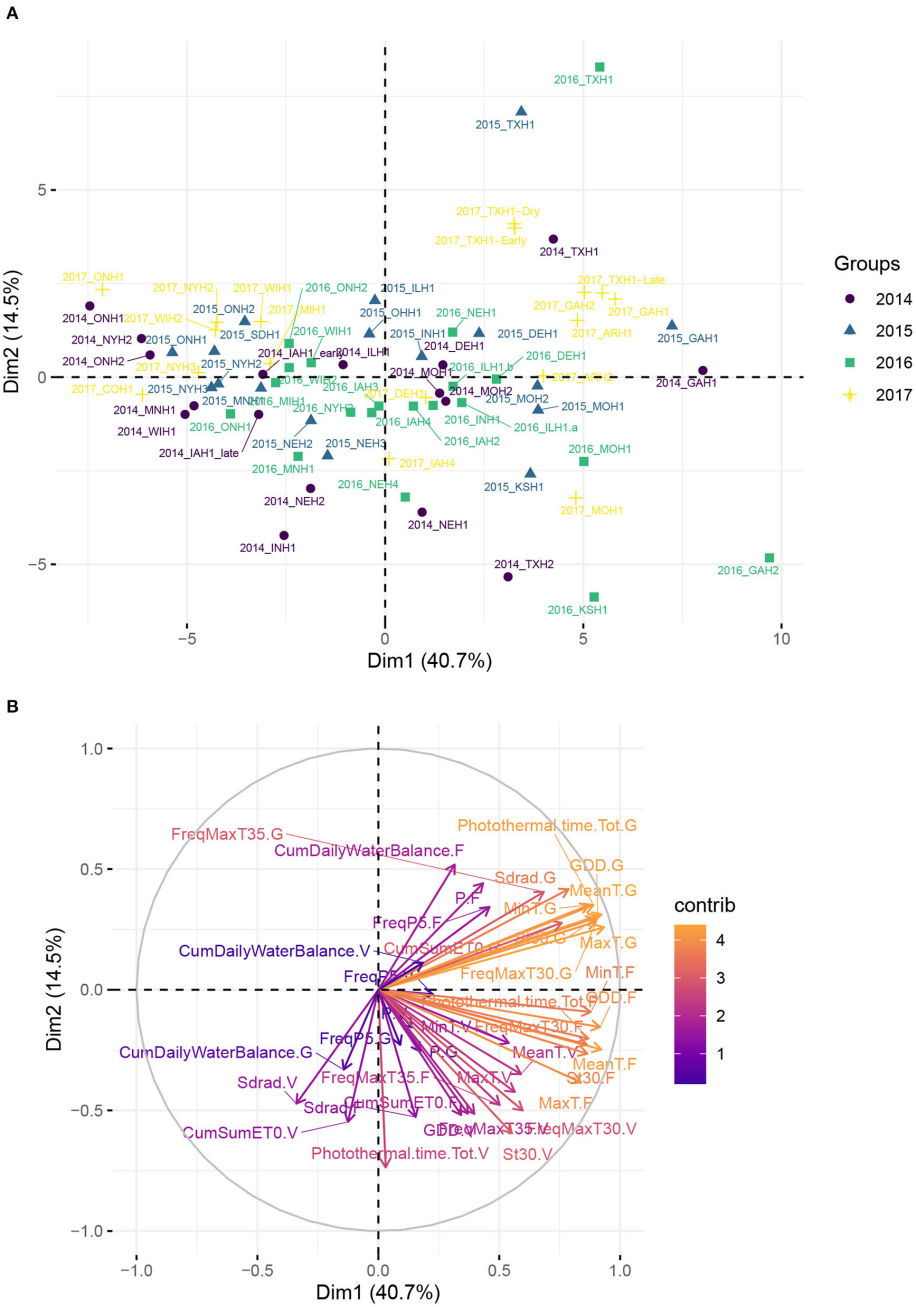
Principal component analysis (PCA) plot of environmental data from the 71 environments, using the median flowering date as reference in each environment. **(A)** Maize trial experiments located in the US and in Canada used in analyses. Name of the locations and their geographical position are given in Supplementary Table 1. **(B)** Correlation plot of the weather-based covariates used in the PCA.

Figure 2 reveals a partitioning of environments into clusters corresponding mostly to different US climate zones. It suggests that the sample of environments was broad enough to cover a large spectrum of environmental conditions across the North-American continent. The first two principal components explained more than 55% of total variation among environments on the basis of weather-based environmental covariates. The loading plot shows that MinT.F and GDD.F, FreqMaxT30.G, which are covariates related to temperature during flowering and grain filling stage, strongly influenced the first principal component (PC1). Environments from the South/Southeast (Arkansas, Texas, Georgia) showed positive PC1 and PC2 scores, which can be explained by a common humid subtropical climate, according to the Köppen climate type classification (Köppen and Geiger, 1930). One exception was one location in Texas (denoted 2014_TXH2), associated with more semi-arid climatic conditions. These results indicate that a closer geographical distance does not necessarily imply similar environmental conditions, based on climate types. For instance, environments from Delaware were closer to environments from the Midwest than Northeastern environments. Environments from the Midwest, associated with a humid continental climate, were situated mostly around the origin of the plot, and environments further north or in Canada exhibited the lowest temperatures among this set of sampled environments and presented a negative PC1 score.

### 3.2. Hyperparameter Importance for Gradient Boosting Approaches

Computing by fANOVA the marginal contribution of each tuned hyperparameter, using the performance data gathered during the hyperparameter optimization step on the different training sets, highlights large differences regarding their respective impact on model performance (Supplementary Figure 3). For the two gradient boosting algorithms, the learning rate (named eta in XGBoost) and the maximum depth of the tree were the most relevant algorithm parameters, as well as their interaction. The number of boosting iterations did not play a major role in model performance. We also found an advantage of using the hyperparameter feature_fraction and colsample_bytree, implemented in LightGBM and XGBoost, respectively, as it allowed an important reduction of the training time without having any observed negative effect on the accuracy of the predictions. It should be emphasized that we did not fully explore the influence of all possible hyperparameters implemented in these algorithms because of computational limitations, and therefore many of these were fixed during the hyperparameter optimization step.

### 3.3. Comparison of Model Performance Across Two Traits and Four Different CV Scenarios

**CV0-Year**

**Figure 3:**
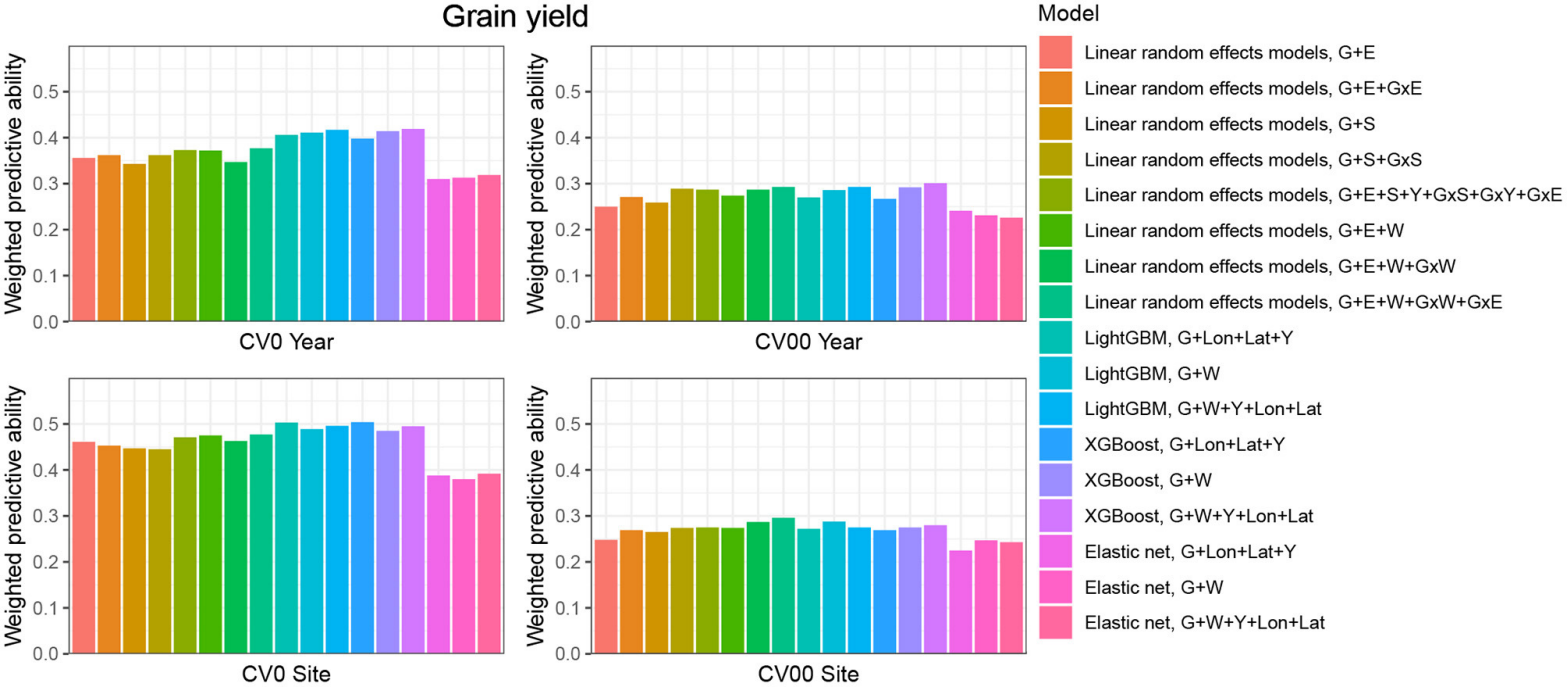
Weighted average predictive ability across 71 environments obtained for four cross-validation schemes and 16 models for the trait grain yield. G, main effect of SNPs markers (genomic relationship matrix for LRE models; principal components derived from marker matrix for machine learning-based approaches); Y, year effect; S, site effect; GxS, genotype-by-site interaction; E, environment effect; GxY, genotype-by-year interaction; GxS, genotype-by-site interaction; GxE, genotype-by-environment interaction; GxW, interaction between W and SNPs; Lon, longitude; Lat, latitude; W, effect of weather- and soil-based covariates. For linear random effects models, results with models including longitude and latitude data in the matrix W are depicted here.

**Figure 4:**
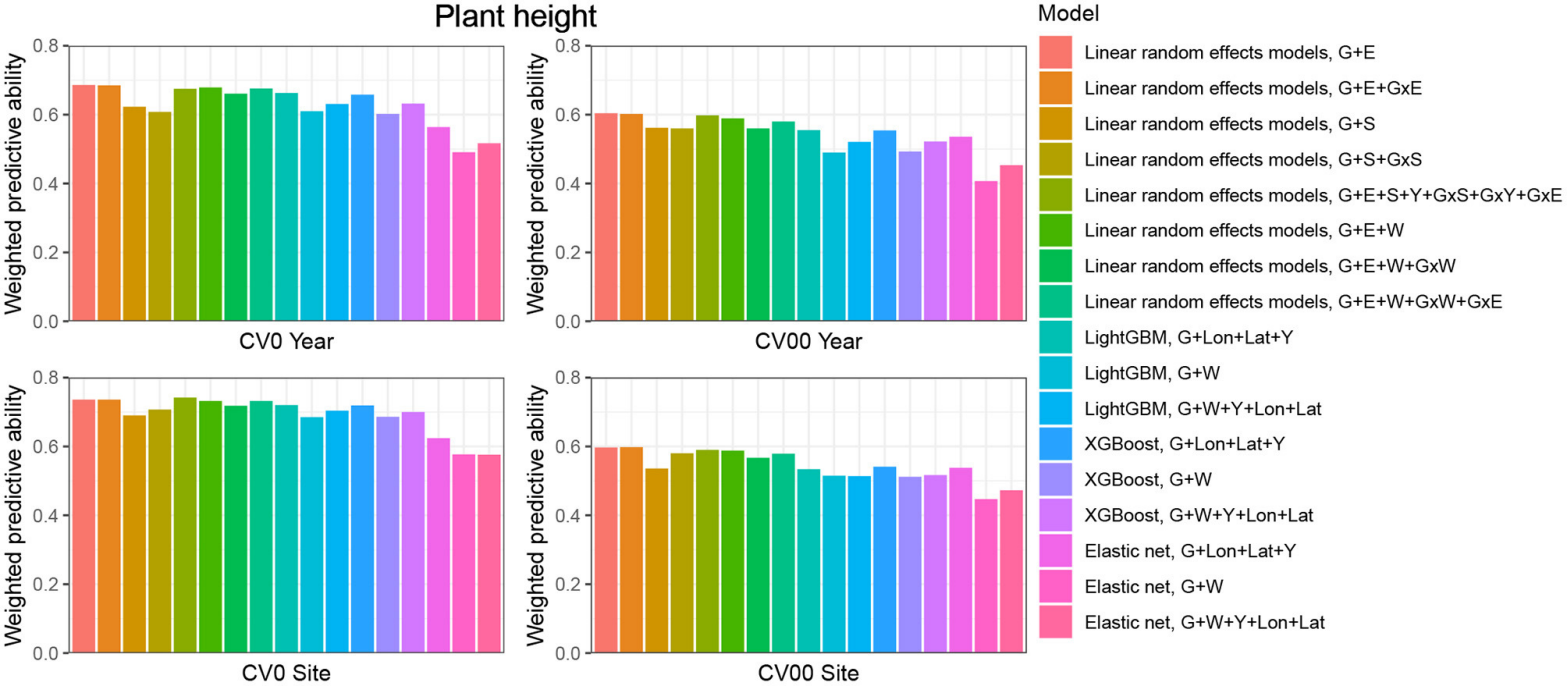
Weighted average predictive ability across 71 environments obtained for four cross-validation schemes and 16 models for the trait plant height. G, main effect of SNPs markers (genomic relationship matrix for LRE models; principal components derived from marker matrix for machine learning-based approaches); Y, year effect; S, site effect; GxS, genotype-by-site interaction; E, environment effect; GxY, genotype-by-year interaction; GxS, genotype-by-site interaction; GxE, genotype-by-environment interaction; GxW, interaction between W and SNPs; Lon, longitude; Lat, latitude; W, effect of weather- and soil-based covariates. For linear random effects models, results with models including longitude and latitude data in the matrix W are depicted here.

**Figure 5:**
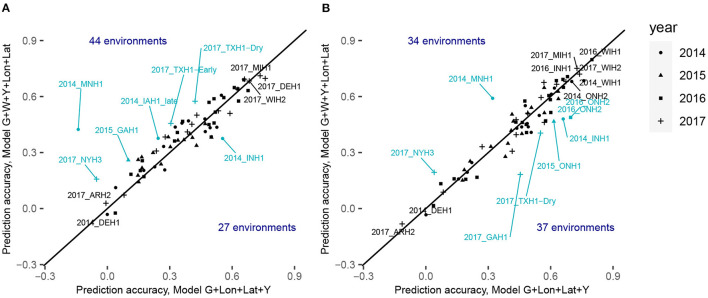
Comparison of the within-environment predictive ability with different sets of predictors for the trait grain yield for XGBoost **(A)** with the CV0-Year scenario and **(B)** CV0-Site scenario. The x-axis corresponds to the within-environment correlation obtained with the model incorporating PCs derived from SNPs, year and geographical coordinates. The y-axis corresponds to the within-environment correlation obtained with the model incorporating PCs, year, W (i.e., weather- and soil-based covariates) and geographical coordinates. The line indicates the identity. Blue-colored points with a label indicate environments for which the absolute difference between the two predictive abilities was superior to 0.13. Black-colored points with a label indicate the least and the most accurately predicted environments.

When the aim was to predict yield performance of already tested hybrids in new environments, the weighted average correlation of the baseline LRE model (G+E) was 0.356 (Figure 3; Supplementary Table 6). When the GxE term was added, the average correlation improved to 0.362. The model that included all interactions (G+E+W+GxW+GxE) was the best LRE model, while using only interactions between environmental covariates and genomic information (model G+E+W+GxW) slightly decreased the predictive ability of the baseline model to 0.347. In this prediction scenario, the two GBDT methods outperform all LRE models; model XGBoost-G+W+Y+Lon+Lat improved upon the baseline model by 18%. In addition, a small increase of predictive ability could be observed when environmental covariates were included as features for the machine learning-based frameworks. Furthermore, models that included geographical coordinates as predictor variables resulted in better prediction accuracies, and this revealed true across all prediction problems; therefore, Figures 4, 5 display results from LRE models using W as including longitude and latitude as predictor variables. For plant height, the baseline model performed best (Figure 4; Supplementary Table 8), and gradient boosting models incorporating environmental predictor variables performed consistently worse than models based only on genotypic data, geographical data and year information.

**CV00-Year**

CV00-Year produced lower average correlation coefficients for the two traits and for all models compared to CV0-Year, which illustrates that genomic prediction in multi-environment trials achieves better results when the training set includes information from the same genotypes evaluated in other environments. Regarding the trait grain yield (Figure 3; Supplementary Table 6), modeling the effect of sites instead of environments resulted in a small improvement of the predictive ability (4% better than the G+E model). Adding the GxE term to the LRE baseline model also positively affected the predictive ability (8% better than the G+E model). However, the LRE model with main site and genotype-by-site interaction effects (G+S+GxS) outperformed LRE models based on the modeling of year-location (E) effects. Overall the best predictive model for this trait was again the GBDT model XGBoost-G+W+Y+Lon+Lat, which displayed an average correlation of 0.301 (20% higher than the baseline model). GBDT models incorporating W performed between 6 and 13% better than GBDT models excluding W, which demonstrates the usefulness of environmental data for prediction of yield performance of new genotypes in an untested year. Among LRE models, the LRE model with all interactions and using enviromental data was the best model and resulted in an improvement of 17% over the baseline model. Regarding the trait plant height (Supplementary Table 8), the best predictive model was the baseline LRE model with an average weighted correlation of 0.604. Among LRE and GBDT models, models which did not include any environmental data performed better than those using these. An explanation for this lack of improvement with environmental data for plant height in this prediction problem can be that year and geographical position are appropriate and sufficient data to efficiently characterize environments for prediction of plant height, while using all environmental variables might generate noise here.

**CV0-Site**

The prediction of already tested genotypes in all environments associated with a common site revealed higher predictive abilities than with the CV0-Year prediction problem (Figures 3, 4; Supplementary Tables 7, 9). Indeed, based on our dataset, which covers many different sites across the US (see Supplementary Figure 1), the leave-one-site-out CV strategy generates large ratios across all training/test splits. This greater amount of data available to predict environments from one site can explain why this CV scheme obtained higher predictive abilities than the CV0-Year strategy. For the trait grain yield (Figure 3; Supplementary Table 7), the XGBoost-G+Lon+Lat+Y outperformed other models, showing an increase of 9% compared to the baseline LRE model. LightGBM models showed also better predictive abilities than LRE models. Only for LRE models did the use of environmental data yield a very small increase in predictive ability; the best result within this type of statistical approach was obtained by the model including all interactions (0.477, 3% higher than the baseline model). However, for the trait plant height (Figure 4; Supplementary Table 9), LRE models performed better than machine learning-based methods, with the model G+E+S+Y+GxS+GxY+GxE, which uses only basic information on environments, showing a mean correlation of 0.742. LightGBM and XGBoost methods with geographical and year information predicted reasonably well compared to the latter model (average r between 0.7 and 0.72), and again, the addition of environmental covariates decreased the predictive ability of GBDT models G+Lon+Lat+Y.

**CV00-Site**

As expected, the prediction of new genotypes in new sites resulted in lower mean correlations than CV0-Site for the two traits under study across predictive models. This highlights again the importance of the relationship between training and test sets. For the trait grain yield (Figure 3; Supplementary Table 7), the weighted average predictive ability of the reference model (G+E) was 0.248, and the model using sites instead of environment main effect was slightly better with a mean correlation of 0.265 (7% over G+E model). When the GxE term was added to the baseline model, the weighted average predictive ability was improved to 0.269 (8% over G+E model). It is worth to underline that models incorporating genotype-by-site effects performed even better (10% and 11% higher than the reference model). Modeling the interaction between ECs and genotypes and between environments and genotypes (model G+E+W+GxW+GxE) yielded an improvement of the baseline model by 19% (average r = 0.296), which was closely followed by the LightGBM and XGBoost models incorporating environmental covariates (between 11 and 16 % increase over the baseline model). As for the CV0-Year and CV00-Year CV schemes, the use of environmental data slightly increased the average predictive ability for grain yield. For the trait plant height (Figure 4; Supplementary Table 9), the baseline model with interactions by environment (G+E+GxE) outperformed other models. As for the previous prediction problems, environmental data decreased predictive abilities over all implemented models for the trait plant height.

When comparing the predictive abilities across traits, grain yield was the trait showing the lowest predictive ability across all CV schemes. Across all CV schemes, Elastic Net was the worst predictive modeling approach, which can be related to the absence of interactions between predictors in this model, if these are not explicitly provided as new features.

Figure 5; Supplementary Tables 10, 11 display the detailed within-environment correlation results for grain yield for two (CV0-Year and CV0-Site) cross-validation schemes. If a predicted environment is over the identity line, this means that there was an increment of the predictive ability by using environmental information. For CV0-Year, the machine learning-based model including environmental data outperformed the model only using geographical and year information in 44 of the 71 considered environments. For CV0-Site, however, the model with environmental features was better than the less complex one in only 34 environments. This can be interpreted as a failure to explain a large part of the GxE by the computed ECs, and by a more efficient representation of environmental effects by simple geographic information.

### 3.4. Variable Importance

**Figure 6:**
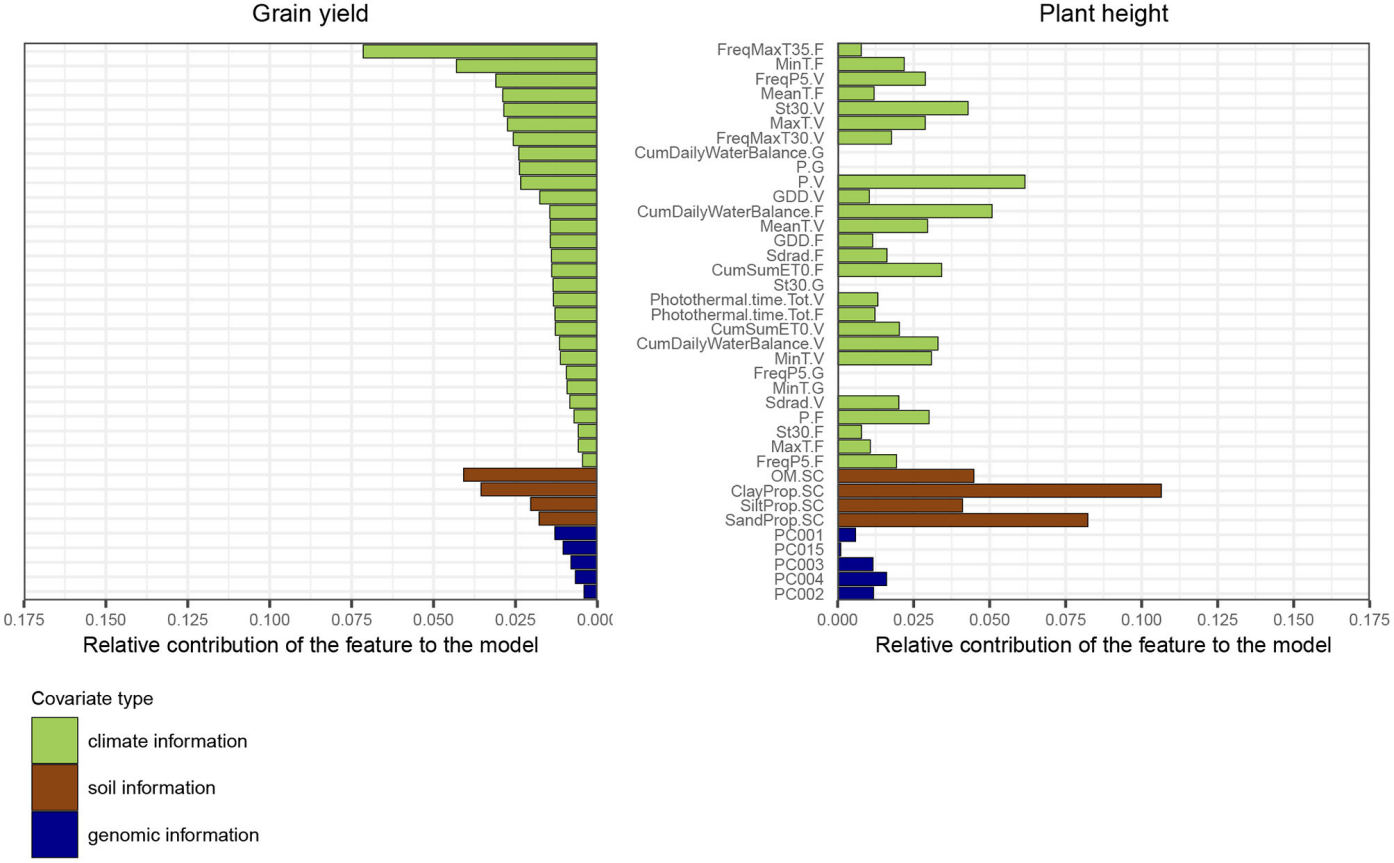
Feature importance ranking based on the average relative gain per feature obtained with the model XGBoost-G+W, for the two traits grain yield and plant height. The metric was estimated using a model fitted on the full dataset. The gain represents the improvement in accuracy when using a feature for splitting, across all trees in the model. The order of features is based on feature performance within covariate class for the trait grain yield. The sum of all feature contributions is equal to 1. Weather-based variables from the grain filling stage were not used to predict plant height.

Regarding the trait grain yield, many of the identified top variables were related to temperature, such as the average minimum temperature during the flowering stage, or the frequency of days during which the maximum temperature was above 35°C (Figure 6). Organic soil matter concentration was the third most important feature, which demonstrates that fields with fertile soils were associated with higher yields. The amount of water received by the field (P.V) during the vegetative and grain filling stage was also a major feature for the model, as well as the frequency of days during the vegetative stage for which the amount of water was greater than 5 mm. Regarding the trait plant height, variables based on soil information played a major role for trait prediction, as they likely affect the crop shoot architecture. The amount of water received during the vegetative stage was also an important explanatory variable for plant height.

**Figure 7:**
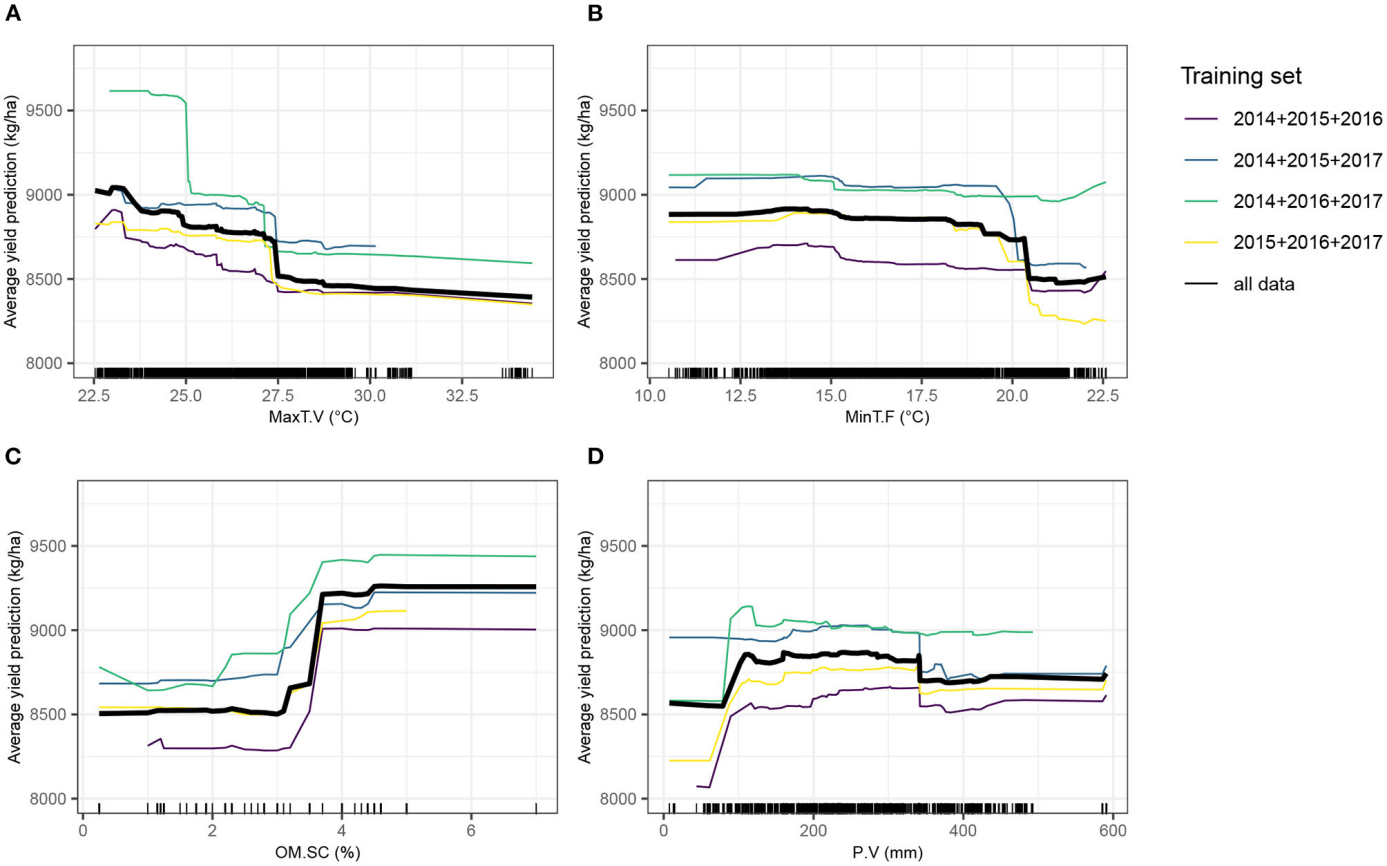
Partial dependence plots (PDPs) showing the behavior of the expected value of predicted yield as a function of four top-ranked predictor variables. The Y-axis value of a PDP is calculated average of all model predictions obtained from the training dataset, when the value of the predictor variable is equal to X. The four training sets from the leave-1-year-out cross-validation scheme (CV0-Year) and the full dataset, separately trained with XGBoost, were used. Tick marks indicate individual observations. **(A)** MaxT.V, maximum temperature during the vegetative stage; **(B)** MinT.F, minimum temperature during the flowering stage; **(C)** OM.SC, percentage of soil organic matter; **(D)** P.V, Amount of precipitation and irrigation during the vegetative stage.

Partial dependence plots (Figure 7) show that minimum temperature at flowering stage was strongly impacting yield from approximately 20°C onwards. Maximum temperature during the vegetative stage had a detrimental effect on yield, suggesting that very elevated temperatures can impair a normal plant growth, eventually required to achieve optimal grain yield, although it tended to have a more gradual effect than minimum temperature at flowering stage. The relationship with yield of the total amount of precipitation during the vegetative stage was positive, before reaching a plateau. A high soil organic matter content yielded in superior yield predicted values.

## 4. Discussion

Breeders, working on the development of climate resilient cultivars, risk making incorrect selection decisions if genotype-by-location and genotype-by-year interactions are not properly accounted for (Jarquín et al., 2017; De Los Campos et al., 2020). By incorporating environmental variables in our models, we assessed the value of these predictor variables for genomic prediction of complex phenotypes across four cross-validation scenarios. Gradient boosting frameworks based on decision trees have demonstrated high prediction performance for traits affected by non-additive effects (Abdollahi-Arpanahi et al., 2020), as well as model interpretability to extract important insights from the model's decision making process (Shahhosseini et al., 2020). Thus, a second objective was to evaluate these new prediction methods on the basis of prediction accuracies and for identification of the most relevant environmental variables.

### 4.1. Comparison of Prediction Methods Across the Two Traits

We observed that GBDT frameworks produced a slightly improved predictive ability for grain yield compared to the linear random effects models in three (CV0-Year, CV00-Year, and CV0-Site) out of the four CV schemes. However, no advantage was observed when GBDT was used to predict plant height. Overall, GBDT methods were competitive to LRE models, but we did not find any case where these machine learning-based methods considerably exceeded the predictive ability of LRE models. Previous studies have suggested that machine learning-based approaches can provide superior accuracy for prediction of phenotypic traits characterized by substantial non-additive effects. For instance, results from Zingaretti et al. (2020) in strawberries suggest that traits, exhibiting large epistatic effects, can be better predicted by convolutional neural networks (CNN), than by Bayesian penalized linear models. On the other hand, for moderately to highly heritable traits, no real advantage of using machine learning-based methods was observed in their study. Bellot et al. (2018) pointed out that human height, a trait with a prevailing additive component and a polygenic architecture, was better predicted by linear methods than by CNNs. For other traits they examined in their study, a deep learning approach did not significantly outperform other methods in terms of prediction accuracy. Similar conclusions were drawn by Azodi et al. (2019) who reported an inconsistency of performance for non-linear machine learning-based algorithms in comparison with linear algorithms, according to the trait under study.

In our study, we incorporated not only genomic-based, but also environmental-based predictor variables. Yield component traits are controlled by numerous physiological processes under the influence of environmental factors, which can explain the large contribution of the GxE variance component for the phenotypic variance of grain yield, while for plant height, the proportion of variance explained by GxE is generally much lower than the proportion of variance related to genetic effects (Olivoto et al., 2017; Rogers et al., 2021). Nonlinear relationships between some environmental factors, such as temperature or rainfall amounts, and grain yield are well-known in the field of ecology and agriculture (Troy et al., 2015; Li et al., 2019). Hence, the slightly better prediction performance for grain yield with GBDT frameworks might originate from their ability to model nonlinear effects of environmental predictor variables, as observed with the partial dependence plots, as well as interactions with other predictor variables like genomic-based principal components. This asset was also described by Heslot et al. (2014b) when implementing soft rule fit (a modified ensemble method) capturing nonlinear interactions between markers and environmental stress covariates. Additional studies are required to validate this hypothesis using other phenotypic traits showing various genetic architectures. Moreover, it should be noted that we used only linear kernels in the reaction norm models to model genetic and environmental similarities. This means that we did not account for the specific combining ability (i.e., nonlinear genetic effects, due to dominance or epistasis, of specific hybrid combinations) which can influence the magnitude of yield heterosis in maize hybrids. Alternative approaches exist to model additive and dominant genetic effects, as well as environmental relatedness with nonlinear kernels (Bandeira e Sousa et al., 2017; Cuevas et al., 2018; Costa-Neto et al., 2020a). Bandeira e Sousa et al. (2017) and Cuevas et al. (2018) obtained better predictive abilities when using a Gaussian kernel rather than a linear GBLUP kernel with multi-environment G–E interactions models. More recently, Costa-Neto et al. (2020a) implemented Gaussian and arc-cosine kernels-based approaches on both genomic and environmental datasets from a MET maize dataset, and noted an improvement in prediction accuracy using these methods across various cross-validation strategies. These results highlight the potential of nonlinear methods to better unravel nonlinear relationships existing in the input space.

### 4.2. Model Performance Under Various Prediction Problems

The four cross-validation schemes we evaluated represent challenging prediction problems. They seeked to assess the ability of the models to predict the effect of unknown combinations of environmental stresses on the studied phenotypic traits in a new year (CV0-Year and CV00-Year) or in a new site (CV0-Site and CV00-Site). Previously published work has revealed somewhat similar ranges of prediction accuracies for this trait in maize (Costa-Neto et al., 2020a; Jarquin et al., 2020). In winter wheat, Jarquín et al. (2017) and Sukumaran et al. (2017) reported the predictions of yield performance in future years (CV0-Year) as the most challenging prediction problem on the basis of results obtained for various cross-validation schemes, and results of Sukumaran et al. (2018) showed that modeling site effect instead of environment effect based on basic information about the environments (year and location) had a positive effect on predictive ability with CV0-Year, as we could also observe for CV0-Year, CV00-Year, and CV00-Site in our results. Indeed, this type of models allows to exploit information from the same site tested across several years. Another factor which is important to take into account in multi-year breeding data, as emphasized by Bernal-Vasquez et al. (2017), is the degree of genetic relatedness between the training and validation sets. Hence, CV00-Year and CV00-Site were more challenging prediction problems than CV0-Year and CV0-Site, respectively, and yielded lower weighted mean correlations across all models.

Regarding the usefulness of environmental information, the best model for grain yield based on mean predictive ability included these data for three (CV0-Year, CV00-Year, and CV00-Site) out of the four CV schemes. In addition, it must be taken into account that much less phenotypic observations were masked for CV0-Site (1/28, about 3.6% on average, with some sites being present more often than others across years in our dataset) than for CV0-Year (1/4, about 25% as the dataset is unbalanced). Hence, we can consider CV0-Year and CV00-Year as more challenging prediction problems than CV0-Site and CV00-Site in our study. The improvement due to the incorporation of environmental data was however less remarkable and less consistent across CV schemes than expected, which was in contrast with previous results. Monteverde et al. (2019) also implemented a leave-1-year-out scenario, with one unique location present in the dataset, and the best prediction accuracies for grain yield were always reached by the models integrating environmental predictors alongside genomic predictors. Findings from Costa-Neto et al. (2020a) also show a significant increase of prediction accuracy with the linear GB kernel incorporating environmental data in a CV0 scheme, but the authors additionally modeled dominant genetic effects, which were not accounted for in our study. On the other hand, Jarquin et al. (2020) also used the same Genomes to Fields dataset and reported a lack of enhancement when using a model that solely incorporated interactions between genotype and environmental covariates (i.e., without using the environment label). The best predictive models for the CV0 and CV00 schemes, that they implemented, included both genotype-by-environment and genotype-by-EC interactions, similarly to our results (Supplementary Tables 6–9). In agreement with the reasons invoked by the authors of this study, we argue that environmental data are especially relevant for predictions when a larger number of environments is used, e.g., by testing sites within a limited geographical range with relatively similar environmental conditions across multiple years. This was for example achieved in the study of De Los Campos et al. (2020), where 16 sites located in France were tested over 16 years. A reasonable hypothesis is that historical weather data obtained across multiple years for a specific geographical area can lend the model reliable information on the effect of year-to-year climatic variation on phenotypic performance, in addition to site-based factors (soil and geographical position). A finding supporting this hypothesis is that the environments, which showed the best prediction accuracies with an environmental model, corresponded generally to the sites which were repeated across years, like Madison (WI) or College Station (TX) (Supplementary Tables 10, 11). Interestingly, 2014_TXH2, a location for which data were only included for a single year, showed a moderate prediction accuracy with the XGBoost model without environmental information in CV0-Year (*r* = 0.28; Supplementary Table 10), which was superior to the model with environmental covariates (*r* = 0.21 with all environmental covariates included). We can suppose that the inclusion of environmental information, when predicting a new environment with properties that are very different from environments covered by the training set, is not useful to enhance the predictive ability of the model using basic predictors, such as the year factor and geographic coordinates. Extreme weather events can make some environments very unpredictable. 2017_ARH1 and 2017_ARH2 exhibited a very low prediction accuracy for grain yield (<0 for 2017_ARH2) in both CV0-Year and CV0-Site (Supplementary Table 11), which is likely to be related to the effect of the tropical storm Harvey at the end of August 2017, which caused substantial lodging due to wind and excessive rainfall affecting the yield, and was reported by collaborators in the metadata.

### 4.3. Incorporation of Weather-Covariates in the Predictive Models

The use of environmental information yielded a small gain in average prediction accuracy for many models tested on grain yield, but did not lead to any improvement for plant height. For this latter trait, the large influence of soil-based variables, illustrated by the variable importance ranking (Figure 6), can also possibly explain why prediction models using only geographical coordinates outperformed more elaborate models. For this trait, latitude and longitude data might indirectly capture information which is site-specific and repeatable across years, e.g., related to the quality of soil. For instance, environments from the Corn Belt, which were present in our dataset, usually exhibited fertile soils with much higher organic soil matter content than environments located in other US regions. Costa-Neto et al. (2020b) highlighted that simple geographic-related information, such as longitude and latitude data, can also efficiently represent environmental patterns that are specific to a site (for instance related to soil characteristics), and hence capture well genotype-by-site interaction while using only two variables.

In general, the lack of real enhancement of predictive ability may result from the way we incorporated developmental stages into our models, as we defined only three main developmental stages (i.e., vegetative, flowering and grain filling stages). Trial data often lack a rigorous collection of phenological data due to phenotyping costs. A possible solution to predict plant developmental stages can be to use crop models, such as APSIM (Holzworth et al., 2014) or SiriusQuality (Keating et al., 2003), as done in related studies (Heslot et al., 2014b; Rincent et al., 2017, 2019; Bustos-Korts et al., 2019). In our case, we did not implement a crop model since we aimed at estimating the flowering stage at the hybrid level as accurately as possible, as it is known to be a critical period for the determination of yield-related components. Therefore, we based our environmental characterization on available field data (sowing date and silking date scored) in order to derive environmental covariates for three main developmental stages, similarly to Monteverde et al. (2019) in rice. The reported variability among crop growth models (CGM) in simulating temperature response can complicate the task of choosing the most appropriate one (Bassu et al., 2014). In addition, the task of integrating genetic variation for earliness in crop growth models can also be rather challenging, with the risk that the predicted developmental crop stages might not appropriately reflect the plant developmental stages observed in the field if the model does not properly account for genotype-specific parameters (Rincent et al., 2019). Technow et al. (2015) developed a complex framework combining both CGM and whole-genome prediction, where the CGM is used to predict grain yield as a function of several physiological traits and of weather and management data. Genotype-specific physiological parameters were estimated in this study by running a Bayesian algorithm which models them as linear functions of the effects of genomic features. It would be of high interest to apply CGM models on this dataset by taking advantage of the flowering time data that are available. We should also mention that other types of input data could be incorporated in future analyses, such as the type of field management, the field disease pressure, preceding crop, or the presence of external treatments (organic, nitrogen fertilizers).

### 4.4. Prerequisites to Use Machine Learning-Based Models and Their Usefulness to Understand Significant Environmental Factors

Specific techniques should be employed to ensure an efficient application of machine learning-based models. These can provide better results when expert knowledge is incorporated (Kagawa et al., 2017; Roe et al., 2020; Brock et al., 2021). Here, we restricted weather information to the duration of the growing season, transformed some raw weather information into new variables (evapotranspiration) and built stress indices besides typical climate covariates based on previous biological knowledge (e.g., detrimental temperature thresholds for maize (Greaves, 1996; Schlenker and Roberts, 2009; Lobell et al., 2014; Zhu et al., 2019; Mimić et al., 2020). Prior understanding of the role of input features can help mitigate the risk of using irrelevant information in the model. As expected, the correlation matrix between environmental covariates (Supplementary Figure 2) showed that numerous predictor variables were highly correlated with each other, especially those related to temperature and heat stress. We did not perform feature selection based on the Pearson correlation coefficients between environmental covariates, because of the risk of dropping highly predictive variables, since the metric ignores the relationship to the output variable. In addition, methods based on decision trees can perform internal feature selection, making them robust to the inclusion of irrelevant input variables and to multicollinearity (Hastie et al., 2009; Kuhn et al., 2013). If two variables are strongly correlated, the decision tree will pick either one or the other when deciding upon a split, which should not eventually affect prediction results. Another approach to reduce the number of features and reduce training time is to apply feature extraction, as we did by deriving principal components from the genotype matrix and use these as new predictor variables in the machine learning-based models. This procedure did not seem to affect model performance.

Machine learning models often require an elaborated hyperparameter optimization strategy, implying for example a nested cross-validation approach which can be computationally expensive (Varma and Simon, 2006), since it involves a series of train/validation/test set splits to prevent data leakage. Inadequate model tuning can result in a suboptimal performance of the algorithm. Here, we found that the hyperparameters such as the learning rate or tree depth were relevant regularization parameters to reduce the model complexity, thereby dealing with overfitting. In accordance with these results, other authors had also reported these two hyperparameters as the most important ones for another gradient boosting library similar to LightGBM, Adaboost (Van Rijn and Hutter, 2018). In general, lower values of the learning rate (<0.01) are recommended to reach the best optimum (Ridgeway, 2007). Nonetheless, as the learning rate is decreased, more iterations are needed to get to the optimum, which implies an increase of the computation time and of additional memory (Ridgeway, 2007; Kuhn et al., 2013). With regard to the tree depth, a relatively low maximal depth generally helped to prevent overfitting, and better results were generally obtained with our data using a tree depth lower than to 8. The deeper a tree is, the more splits it contains, resulting in very complex models which do not generalize well on new data. Knowledge regarding the most important hyperpararmeters to tune is useful if limited computational resources hamper the investigation of numerous hyperparameter combinations during the training phase. Our results demonstrated similar predictive abilities of LightGBM and XGBoost, with a clear speed advantage for LightGBM, which ran often more than twice as fast. This asset relies in particular on a feature implemented in LightGBM, the gradient-based one-side sampling method (GOSS), which implies that not all data actually contribute equally to training. Training instances with large training error (i.e., larger gradients) should be re-trained, while data instances with small gradients are closer to the local minima and indicate that data is well-trained. Hence, this new sampling approach focuses on data points with large gradients and keeps them, while randomly sampling from those with smaller gradient values. A drawback of this method is the risk of biased sampling which might change the distribution of data, but this issue is mitigated in LightGBM by increasing the weight of training instances with small gradients. The main advantage is that it makes LightGBM much faster with comparable accuracy results. Another crucial aspect when applying machine learning models is the adequacy of the dataset for machine learning applications, which should be large enough to allow the algorithm to learn from the data (Géron, 2019). In our case, we benefited from a very large training dataset and a low feature-to-instance ratio (316/18,325).

In our study, on top of prediction applications, tree-based methods were also used to obtain estimates of feature importance, and thereby contributed to a better understanding of key abiotic factors driving the response of the tested genotypes. Feature importance rankings and partial dependence profiles showed that the minimal temperatures and indices related to prolonged heat stress, or to amounts of water received in the field, especially at the flowering stage, ranked among the most important variables for grain yield. When comparing these results with established agronomic knowledge, it was reported that, above a certain threshold, high minimum temperature can lead to an increase of the rate of senescence and reduce the ability of the plant to produce grain across many plant species (Hatfield et al., 2011; Hatfield and Prueger, 2015). Previous research also revealed that increases in average night temperatures were associated with a reduction of grain yield in maize (Millet et al., 2019) and in rice (Welch et al., 2010). In an alternative study on rice cultivars in Colombia, Delerce et al. (2016) identified high minimum temperature (above 22.7°C) as one of the most important environmental factors negatively impacting grain yield by using a machine learning approach based on conditional inference trees. Exposure to temperatures exceeding 35°C during the flowering stage was also a key factor in our study (best predictor variable for grain yield), which can be related to a loss of pollen viability, and consequently to a reduced final kernel set (Hatfield et al., 2011). In our study, water availability at vegetative and grain-filling stages appeared to affect yield, in accordance with the literature outlining that any water deficit during these growth stages can impact grain yield (Denmead and Shaw, 1960; Cakir, 2004), with a more significant impact when water stress occurs during the grain-filling stage (Cakir, 2004). Caution should nonetheless be taken regarding feature importance ranking due to the important correlations between some environmental variables. Furthermore, only 4 years of field trials were used in our analyses, therefore variable importances could be refined with additional data from following years, to mitigate the influence of some environments characterized by adverse climatic conditions and potentially acting as outliers.

### 4.5. Applications

The usefulness of medium to high prediction accuracies, when predicting the performance in a new environment, must always be related to our predictability of the environmental variation. If the weather fluctuates considerably year to year, then the environmental predictors used to compute these predictions might be very different from the true value in the corresponding year. In addition, even if more precise climate change models were available to improve upon the precision of environmental predictors, predictions of observations falling outside the applicability domain, i.e., the range of predictor space in the training set for which the model can give relativey accurate predictions (Netzeva et al., 2005), might not be trustworthy and should be used cautiously (Kuhn et al., 2013). The degree of similarity of the new test set to the training set should hence always be carefully considered.

While some environmental factors are repeatable from year to year, such as the soil type or agronomic practices, a large part of the GxE variation is attributable to weather patterns. Hence, the success of this type of prediction scenario depends on the relative stability of the climate in the targeted regions across years. Nonetheless, we posit that our approach presents two key advantages to predict performance in future years. First, because they are fundamentally data-directed, the tree-based models can take into account new phenotypic data in the training set in a more flexible manner than classical mixed models, without the need to explicitly specify interactions for example. The development of high-throughput phenotyping technologies announces a future enhancement of rapid and accurate training data (Juliana et al., 2019). The predictive frameworks we presented here can make use of new information to refine the estimated effects of the predictor variables. Secondly, we were able to predict a quantitative phenotype in a new environment by using a novel configuration of genotypic and environmental predictors describing it. A point of interest relates to resource allocation and the possibility to select more efficiently candidates to test in field trials. Based on the exploration of different plausible climatic scenarios—within a range of conditions experienced by the training set—these models can help to evaluate which genotypes might be more adapted to which range of environmental conditions. For regions or target population of environments presenting relatively stable climatic conditions across years, the probability of success of this type of predictive modeling approach is heightened.

## 5. Conclusions

Encouraged by the effectiveness of machine learning-based frameworks reported in the recent literature across various research fields, we compared two popular ensemble models with linear random effects models implemented in a Bayesian framework and a regularized linear model. In three CV schemes with the trait grain yield, the use of gradient boosting models resulted in a slight improvement of the average predictive ability but not for plant height. This finding indicates that machine learning-based approaches can be envisaged for genomic prediction but their efficiency may vary according to the trait under study and its degree of responsiveness to environmental variation. For a trait strongly under the influence of environmental factors, machine learning-based models could provide predictive abilities similar or slightly superior to linear random effects, and could additionally be used for interpretation of feature ranking and to build partial dependence plots detailing relationships between predictor variables and outcome. Provided further efficiency gains in machine learning algorithms, as well as the standardization and harmonization of large-scale environmental data, new opportunities in the field of predictive modeling for developing climate resilient varieties appear forthcoming.

## Data Availability Statement

Publicly available datasets were analyzed in this study. Raw genotypic, phenotypic, weather, and soil data from the Genomes to Fields Initiative can be found at: https://datacommons.cyverse.org/browse/iplant/home/shared/commons_repo/curated/GenomesToFields_2014_2017_v1.

## Author Contributions

CW analyzed the data and wrote the manuscript. TB and HS supervised research. CW, TB, HS, GM, and PT designed the study. TB, HS, GM, SdS, and PT supported with statistical advice. CW, TB, HS, GM, SdS, PT, MS, and J-CR participated in the interpretation of results and contributed to discussion. All authors contributed to the writing of the final draft and approved the manuscript.

## Funding

Financial support for CW was provided by KWS SAAT SE by means of a Ph.D. fellowship. Additional financial support was provided by the University of Göttingen and by the Center for Integrated Breeding Research. We acknowledge support by the Open Access Publication Funds of the Göttingen University.

## Conflict of Interest

The authors declare that the research was conducted in the absence of any commercial or financial relationships that could be construed as a potential conflict of interest.

## Publisher's Note

All claims expressed in this article are solely those of the authors and do not necessarily represent those of their affiliated organizations, or those of the publisher, the editors and the reviewers. Any product that may be evaluated in this article, or claim that may be made by its manufacturer, is not guaranteed or endorsed by the publisher.
