# Supplementary material for: Prediction of Maize Phenotypic Traits With Genomic and Environmental Predictors Using Gradient Boosting Frameworks
Source: Front Plant Sci. 2021 Nov 11;12:699589. doi: 10.3389/fpls.2021.699589 (PMC8647909; doi:10.3389/fpls.2021.699589)
Supplement: Supplementary file 1 [file Data_Sheet_1.PDF]

Supplementary Material

1 SUPPLEMENTARY TABLES AND FIGURES

1.1 Figures

Figure S1: Maps of the experimental trials used in this study (from original Genomes To Fields Initiative datasets). Sample size designates the number of phenotypic observations for grain yield. Some points gather several experiments at very close distance from each other.

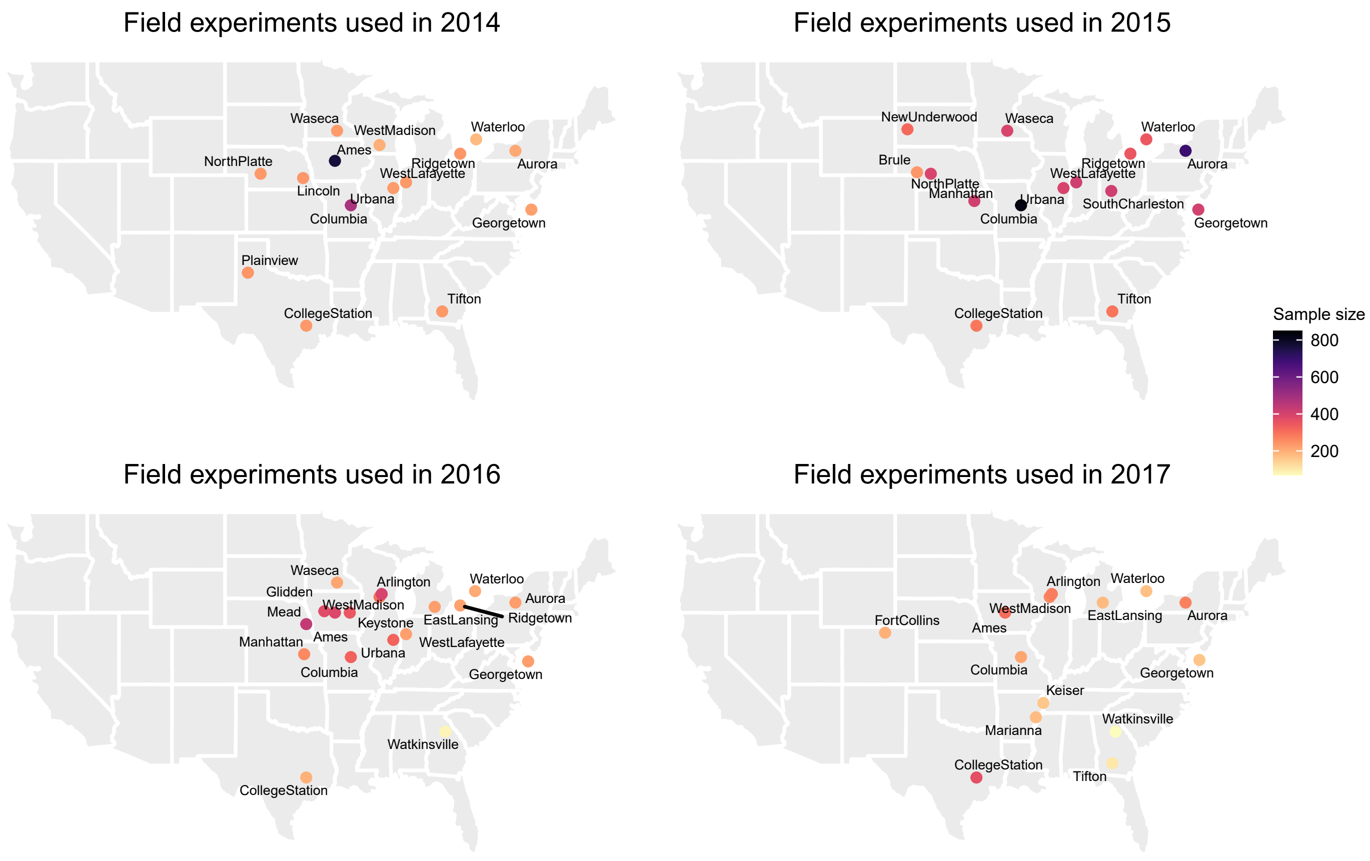

Figure S2: Pearson's coefficients of correlation within and across weather- and soil-based environmental predictors across 71 environments and 18,325 phenotypic observations. Nonsignificant coefficients (P < 0.01) were left blank; please refer to Table 1 for the abbreviations of the environmental predictors.

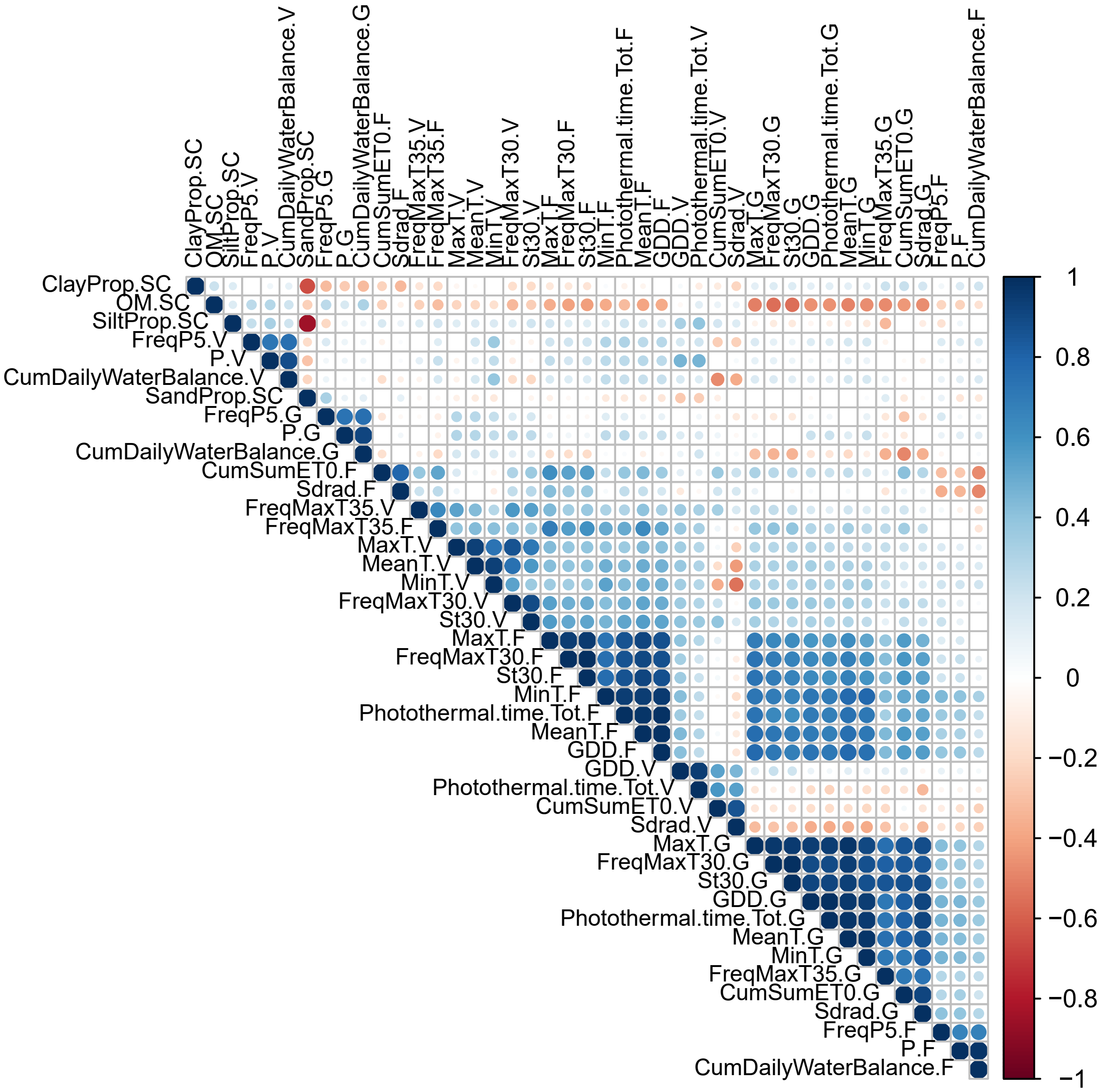

Figure S3: Boxplots showing the most important hyperparameters and interactions between them for **(A)** LightGBM and **(B)** XGBoost, using performance metrics obtained in the leave-one-year-out CV schemes (CV0-Year and CV00-Year). Hyperparameter importance values were obtained from fitting a random forest model on performance data obtained with various hyperparameter settings, followed by a functional ANOVA analysis.

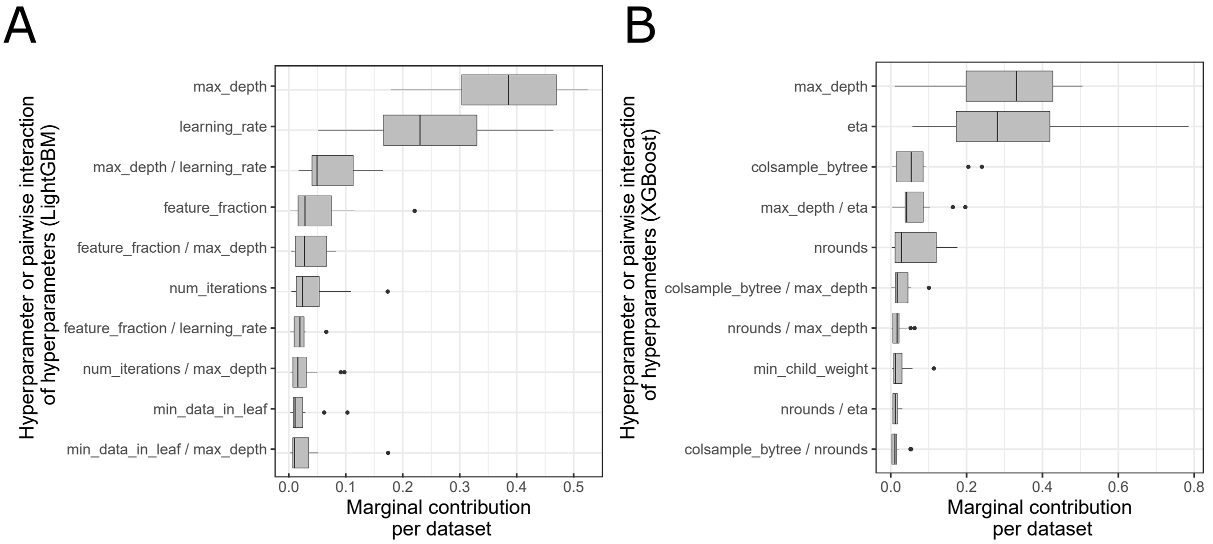

Table S1: Seventy-one trial experiments from the Genomes to Fields datasets used in analyses, with the location, the university factor (used to determine the training/test splits in the CV0-Site and CV00-Site CV schemes), longitude (Lon), latitude (Lat), the planting and harvest dates (in day of year) and the irrigation status of the field experiment. Not all of the environments originally present in the Genomes to Fields datasets were eventually incorporated in the prediction analyses (see Table S2).

ID_Experiment	Location, US State	Site/University factor	Lon	Lat	Planting Date (DOY)	Harvest Date (DOY)	Irrigated (and tracked)	number of yield records used	number of plant height records used
2014_DEH1	Georgetown1, DE	UniversityDelaware	-75.20	38.64	125	272	yes	224	224
2014_GAH1	Tifton, GA	UniversityGeorgia1	-83.56	31.51	94	254	yes	237	236
2014_IAH1_early	Ames, IA	IowaStateUniversity1	-93.70	42.00	129	293	no	131	131
2014_IAH1_late	Ames, IA	IowaStateUniversity1	-93.70	42.00	137	294	no	634	619
2014_ILH1	Urbana, IL	UniversityIllinois	-88.23	40.06	126	280	no	233	229
2014_INH1	WestLafayette, IN	PurdueUniversity	-87.01	40.49	145	322	no	237	236
2014_MNH1	Waseca, MN	UniversityMinnesota	-93.53	44.07	136	289	no	232	230
2014_MOH1	Columbia, MO	UniversityMissouri	-92.21	38.90	127	295	yes	244	243
2014_MOH2	Columbia HinksonCreek, MO	UniversityMissouri	-92.35	38.93	125	317	no	240	239
2014_NEH1	Lincoln, NE	UniversityNebraska	-96.66	40.83	136	295	no	242	242
2014_NEH2	North Platte, NE	UniversityNebraska3	-100.75	41.05	135	309	no	237	234
2014_NYH2	Aurora, NY	CornellUniversity	-76.65	42.73	148	336	no	212	208
2014_ONH1	Waterloo, ON	GuelphUniversity2	-80.43	43.50	139	308	no	181	179
2014_ONH2	Ridgetown, ON	GuelphUniversity1	-81.88	42.45	147	333	no	240	239
2014_TXH1	CollegeStation, TX	TexasAMUniversity1	-96.43	30.55	60	233	yes (not tracked)	236	235
2014_TXH2	Plainview, TX	TexasAMUniversity2	-101.95	34.18	113	273	yes (not tracked)	243	242
2014_WIH1	WestMadison, WI	UniversityWisconsin1	-89.53	43.06	129	301	no	200	197
2015_DEH1	Georgetown2, DE	UniversityDelaware	-75.47	38.63	119	257	yes	407	407
2015_GAH1	Tifton, GA	UniversityGeorgia1	-83.56	31.51	91	238	yes	295	294
2015_ILH1	Urbana, IL	UniversityIllinois	-88.23	40.06	120	275	no	393	392

2015_INH1	WestLafayette, IN	PurdueUniversity	-87.00	40.48	134	288	no	414	414
2015_KSH1	Manhattan1, KS	KansasStateUniversity	-96.61	39.22	113	264	no	408	
2015_MNH1	Waseca, MN	UniversityMinnesota	-93.53	44.07	139	314	no	395	394
2015_MOH1	Columbia, MO	UniversityMissouri	-92.21	38.90	124	279	no	411	410
2015_MOH2	Columbia, MO	UniversityMissouri	-92.21	38.90	125	275	no	411	409
2015_NEH2	North Platte, NE	UniversityNebraska3	-100.75	41.05	113	299	no	394	
2015_NEH3	Brule, NE	UniversityNebraska2	-101.99	41.16	161	348	no	242	
2015_NYH2	Aurora, NY	CornellUniversity	-76.65	42.73	127	320	no	347	344
2015_NYH3	Aurora, NY	CornellUniversity	-76.66	42.72	143	322	no	348	348
2015_OHH1	South Charleston, OH	OhioStateUniversity	-83.66	39.86	141	290	no	413	412
2015_ONH1	Waterloo, ON	GuelphUniversity2	-80.45	43.50	120	288	no	345	345
2015_ONH2	Ridgetown, ON	GuelphUniversity1	-81.88	42.45	127	285	no	350	349
2015_SDH1	New Underwood, SD	SouthDakotaUniversity	-102.93	44.21	142	301	no	317	316
2015_TXH1	College Station, TX	TexasAMUniversity1	-96.43	30.55	66	209	no	290	287
2016_DEH1	Georgetown2, DE	UniversityDelaware	-75.45	38.65	116	258	no	227	225
2016_GAH2	Watkinsville, GA	UniversityGeorgia2	-83.31	33.72	146	286	no	84	84
2016_IAH2	Glidden, IA	IowaStateUniversity2	-94.73	42.07	116	285	no	375	372
2016_IAH3	Keystone, IA	IowaStateUniversity3	-92.26	41.99	115	280	no	352	351
2016_IAH4	Ames, IA	IowaStateUniversity1	-93.70	42.00	117	291	no	388	384
2016_ILH1.a	Urbana, IL	UniversityIllinois	-88.23	40.06	127	283	no	125	125
2016_ILH1.b	Urbana, IL	UniversityIllinois	-88.23	40.06	117	283	no	201	201
2016_INH1	WestLafayette, IN	PurdueUniversity	-86.99	40.48	140	280	no	224	221
2016_KSH1	Manhattan2, KS	KansasStateUniversity	-96.63	39.14	106	271	no	262	262
2016_MIH1	EastLansing Powrline, MI	MichiganStateUniversity	-84.30	42.41	145	321	no	227	227
2016_MNH1	Waseca, MN	UniversityMinnesota	-93.53	44.07	138	294	no	216	
2016_MOH1	Columbia, MO	UniversityMissouri	-92.21	38.89	144	281	no	328	326

2016_NEH1	Mead, NE	UniversityNebraska4	-96.42	41.17	127	313	no	225	225
2016_NEH4	Mead, NE	UniversityNebraska4	-96.42	41.17	159	314	no	211	211
2016_NYH2	Aurora, NY	CornellUniversity	-76.66	42.73	131	343	no	228	226
2016_ONH1	Waterloo, ON	GuelphUniversity2	-80.45	43.50	125	289	no	207	206
2016_ONH2	Ridgetown, ON	GuelphUniversity1	-81.88	42.45	132	306	no	223	222
2016_TXH1	CollegeStation, TX	TexasAMUniversity1	-96.43	30.55	64	218	no	197	193
2016_WIH1	WestMadison, WI	UniversityWisconsin1	-89.53	43.06	130	288	no	303	300
2016_WIH2	Arlington, WI	UniversityWisconsin2	-89.34	43.33	145	299	no	395	392
2017_ARH1	Marianna, AR	ArkansasStateUniversity1	-90.76	34.73	107	254	yes	178	175
2017_ARH2	Keiser, AR	ArkansasStateUniversity2	-90.07	35.67	115	259	yes	160	159
2017_COH1	FortCollins, CO	ColoradoStateUniversity	-105.00	40.65	151	326	no	199	195
2017_DEH1	Georgetown2, DE	UniversityDelaware	-75.43	38.67	118	251	no	163	162
2017_GAH1	Tifton, GA	UniversityGeorgia1	-83.56	31.51	94	250	no	107	106
2017_GAH2	Watkinsville, GA	UniversityGeorgia2	-83.30	33.73	122	251	no	72	72
2017_IAH4	Ames, IA	IowaStateUniversity1	-93.69	41.99	127	290	no	310	307
2017_MIH1	EastLansing, MI	MichiganStateUniversity	-84.49	42.68	142	293	no	180	179
2017_MOH1	Columbia, MO	UniversityMissouri	-92.20	38.89	135	292	no	217	216
2017_NYH2	Aurora, NY	CornellUniversity	-76.65	42.73	138	328	no	187	185
2017_NYH3	Aurora, NY	CornellUniversity	-76.65	42.73	138	328	no	88	88
2017_ONH1	Waterloo, ON	GuelphUniversity2	-80.43	43.50	137	304	no	171	169
2017_TXH1-Dry	CollegeStation, TX	TexasAMUniversity1	-96.43	30.55	62	206	no	112	111
2017_TXH1-Early	CollegeStation, TX	TexasAMUniversity1	-96.43	30.55	62	212	no	146	146
2017_TXH1-Late	CollegeStation, TX	TexasAMUniversity1	-96.43	30.55	96	222	no	109	107
2017_WIH1	WestMadison, WI	UniversityWisconsin1	-89.53	43.06	125	292	no	266	261
2017_WIH2	Arlington, WI	UniversityWisconsin2	-89.34	43.32	131	310	no	279	276

Table S2: List of discarded environments from the original G2F phenotypic datasets, with the reason justifying their removal

Year_Exp	Reason
2014_IAH2	no flowering date
2014_IAH3	no flowering date
2014_IAH4	no flowering date
2014_NCH1	no flowering date
2014_NEH3	no flowering date
2014_NYH1	no silking date
2015_NCH1	no flowering date
2015_NEH1	no silking date
2015_NEH4	no silking date
2015_NYH1	disease field treatment
2015_TXH2	no location nor geographical coordinates in metadata file
2015_WIH1	no flowering date
2015_WIH2	no flowering date
2016_ARH1	many recorded dates with field irrigation but no amount tracked in the metadata
2016_ARH2	many recorded dates with field irrigation but no amount tracked in the metadata
2016_GAH1	many recorded dates with field irrigation but no amount tracked in the metadata
2016_IAH1	no flowering date
2016_NCH1	no flowering date
2016_NYH1	disease field treatment
2016_NYH3	no yield data
2016_OHH1	no flowering date
2016_SCH1	no yield data
2016_TXH2	no location nor geographical coordinates in metadata file
2017_IAH1	no flowering date
2017_IAH2	no flowering date
2017_IAH3	no flowering date
2017_ILH1	no location nor geographical coordinates in metadata file
2017_INH1	no location nor geographical coordinates in metadata file
2017_MNH1	based on comments regarding low phenotypic quality from metadata file
2017_NCH1	no flowering date
2017_NEH3	no flowering date
2017_NEH4	no flowering date
2017_NYH1	disease field treatment
2017_OHH1	no flowering date
2017_ONH2	strange values for flowering date (FW 30 days after sowing date?)
2017_SCH1	no yield data
2017_TXH2	no location nor geographical coordinates in metadata file

Table S3. Quality control applied to daily and semi-hourly weather data. Data which did not meet the specified requirements were flagged and assigned as missing values.

Validation procedure	Air temperature (°C)	Precipitation (mm)	Relative humidity (%)
Range test	-40 $\leq$ T $\leq$ 60	0 $\leq P_{sh} \leq$ 120 (Estévez et al., 2011) 0 $\leq$ P $\leq$ 250	0 $\leq$ RH $\leq$ 100
Persistence tests	var(T_{sh}) $\neq$ 0		var(RH_{sh}) $\neq$ 0
Number of records per day	n(T_{sh})= {24,48,72,96}	n(P_{sh})= {24,48,72,96}	n(RH_{sh})= {24,48,72,96}
Percentage of missing data	r(T) $\geq$ 0.9	r(P) $\geq$ 0.9	r(RH) $\geq$ 0.9
Internal consistency tests	$T_{max} \geq T_{mean} \geq T_{min}$ T_{max}(d) $\geq T_{min}$(d-1) T_{min}(d) $\leq T_{max}$(d-1) (Estévez et al., 2011)		$RH_{max} \geq RH_{mean} \geq RH_{min}$ (Estévez et al., 2011)

T: daily or semi-hourly temperature (°C); T_{sh}: semi-hourly temperature (°C); T_{max}: daily maximum temperature (°C); T_{min}: daily minimum temperature (°C); T_{mean}: daily average temperature (°C); n(T_{sh}): total number of temperature records per day; r(T): ratio of non-missing semi-hourly or hourly temperature records to the total number of temperature records per day; P: daily precipitation (mm); P_{sh}: semi-hourly precipitation (mm); n(P_{sh}): total number of temperature records per day; r(P): ratio of non-missing semi-hourly or hourly precipitation records to the total number of precipitation records per day; RH: mean, maximum or minimum daily relative humidity (%); RH_{sh}: semi-hourly relative humidity (%) ; RH_{max} : daily maximum relative humidity (%); RH_{min} : daily minimum relative humidity (%); RH_{mean} : daily mean relative humidity (%); n(RH_{sh}): total number of relative humidity records per day; r(RH): ratio of non-missing semi-hourly or hourly relative humidity records to the total number of relative humidity records per day; d: day d; d-1: day before day d.

Note: exceptions were tolerated regarding the number of daily records throughout the season, if it was shown that the device correctly recorded a sufficient amount of weather data per day. These exceptions were decided on a case-by-case examination.

Table S4. Hyperparameters tuned with Bayesian optimization for regression models implemented.

Algorithm (R package)	Hyperparameter	Meaning	Min	Max
glmnet	alpha	elastic net mixing parameter	0	1
	lambda	penalty	0	1
XGBoost	nrounds	maximum number of iterations	4000	7000
	min_child_weight	minimum number of samples required to create a new node	5	18
	colsample_bytree	subsample fraction of features to use when constructing each tree	0.4	0.8
	max_depth	maximum depth of a tree	2	12
	eta	learning rate	3e-4	0.01
LightGBM	num_iterations	maximum number of iterations	4000	7000
	min_data_in_leaf	minimum number of samples in a leaf	5	18
	feature_fraction	subsample fraction of features to use when constructing each tree	0.4	0.8
	max_depth	maximum depth of a tree	2	12
	learning_rate	learning rate	3e-4	0.01

Table S5. Linear random effects (LRE) models evaluated in four cross-validation scenarios (CV0-Year, CV00-Year, CV0-Site, CV00-Site). E, environment (YearxSite combination); G, SNPs markers;Y, year; S, site; W, environmental covariates + longitude + latitude; GxE, interactions between environments and markers; GxS, interactions between sites and markers; GxY, interactions between years and markers; GxW, interactions between markers and environmental covariates.

Model abbreviation	Effects included								
	Main effects					Interaction terms			
	G	E	S	Y	W	GxE	GxS	GxY	GxW
G+E	X	X							
G+S	X		X						
G+Y	X			X					
G+E+GxE	X	X				X			
G+S+GxS	X		X				X		
G+Y+GxY	X			X				X	
G+E+S+Y+GxS+GxY+GxE	X	X	X	X		X	X	X	
G+W	X				X				
G+E+W	X	X			X				
G+W+GxW	X				X				X
G+E+W+GxW	X	X			X				X
G+E+W+GxW+GxE	X	X			X	X			X

Table S6. Weighted average correlation between predicted and observed values across 71 environments for the trait grain yield for two cross-validation schemes leaving one year out (CV0-Year, CV00-Year) and for four types of statistical models (XGBoost, LightGBM, Elastic net and linear random effects model, i.e. LRE model) tested with different combinations of predictor variables. The best model for each cross-validation scheme is written in bold.

Type of statistical model	Predictors used	Weather- and soil- based variables included	Longitude and latitude included	CV0: Leave-one-year-out	CV00: Leave-one-year-out, new genotypes
XGBoost	G+W+Y+Lon+Lat	Y	Y	**0.419**	**0.301**
XGBoost	G+W	Y	N	0.414	0.292
XGBoost	G+Lon+Lat+Y	N	Y	0.398	0.267
LightGBM	G+W+Y+Lon+Lat	Y	Y	0.417	0.293
LightGBM	G+W	Y	N	0.411	0.286
LightGBM	G+Lon+Lat+Y	N	Y	0.406	0.27
Elastic net	G+W+Y+Lon+Lat	Y	Y	0.319	0.226
Elastic net	G+W	Y	N	0.313	0.231
Elastic net	G+Lon+Lat+Y	N	Y	0.31	0.241
LRE model	G+E	N	N	0.356	0.25
LRE model	G+E+GxE	N	N	0.362	0.271
LRE model	G+S	N	N	0.343	0.259
LRE model	G+S+GS	N	N	0.362	0.289
LRE model	G+Y	N	N	0.32	0.193
LRE model	G+Y+GxY	N	N	0.313	0.199
LRE model	G+E+S+Y+GxS+GxY+GxE	N	N	0.373	0.287
LRE model	G+W	Y	N	0.341	0.256
LRE model	G+E+W	Y	N	0.371	0.273
LRE model	G+W+GxW	Y	N	0.316	0.258
LRE model	G+E+W+GxW	Y	N	0.347	0.281
LRE model	G+E+W+GxW+GxE	Y	N	0.377	0.291
LRE model	G+W	Y	Y	0.35	0.26
LRE model	G+E+W	Y	Y	0.372	0.274
LRE model	G+W+GxW	Y	Y	0.323	0.267
LRE model	G+E+W+GxW	Y	Y	0.347	0.287
LRE model	G+E+W+GxW+GxE	Y	Y	0.377	0.293

Table S7. Weighted average correlation between predicted and observed values across 71 environments for the trait grain yield for two cross-validation schemes leaving one site out (CV0-Site, CV00-Site) and for four types of statistical models (XGBoost, LightGBM, Elastic net and linear random effects model, i.e. LRE model) tested with different combinations of predictor variables. The best model for each cross-validation scheme is written in bold.

Type of statistical model	Predictors used	Weather- and soil- based variables included	Longitude and latitude included	CV0: Leave-one-site-out	CV00: Leave-one-site-out, new genotypes
XGBoost	G+W+Y+Lon+Lat	Y	Y	0.495	0.28
XGBoost	G+W	Y	N	0.485	0.275
XGBoost	G+Lon+Lat+Y	N	Y	**0.504**	0.269
LightGBM	G+W+Y+Lon+Lat	Y	Y	0.496	0.275
LightGBM	G+W	Y	N	0.489	0.288
LightGBM	G+Lon+Lat+Y	N	Y	0.503	0.272
Elastic net	G+W+Y+Lon+Lat	Y	Y	0.392	0.243
Elastic net	G+W	Y	N	0.38	0.247
Elastic net	G+Lon+Lat+Y	N	Y	0.388	0.225
LRE model	G+E	N	N	0.461	0.248
LRE model	G+E+GxE	N	N	0.453	0.269
LRE model	G+S	N	N	0.447	0.265
LRE model	G+S+GS	N	N	0.445	0.274
LRE model	G+Y	N	N	0.392	0.177
LRE model	G+Y+GxY	N	N	0.403	0.185
LRE model	G+E+S+Y+GxS+GxY+GxE	N	N	0.471	0.275
LRE model	G+W	Y	N	0.423	0.264
LRE model	G+E+W	Y	N	0.475	0.274
LRE model	G+W+GxW	Y	N	0.379	0.242
LRE model	G+E+W+GxW	Y	N	0.46	0.287
LRE model	G+E+W+GxW+GxE	Y	N	0.475	0.294
LRE model	G+W	Y	Y	0.437	0.276
LRE model	G+E+W	Y	Y	0.475	0.274
LRE model	G+W+GxW	Y	Y	0.4	0.257
LRE model	G+E+W+GxW	Y	Y	0.463	0.287
LRE model	G+E+W+GxW+GxE	Y	Y	0.477	**0.296**

Table S8. Weighted average correlation between predicted and observed values across 71 environments for the trait plant height for two cross-validation schemes leaving one year out (CV0-Year, CV00-Year) and for four types of statistical models (XGBoost, LightGBM, Elastic net and linear random effects model, i.e. LRE model) tested with different combinations of predictor variables. The best model for each cross-validation scheme is written in bold.

Type of statistical model	Predictors used	Weather- and soil- based variables included	Longitude and latitude included	CV0: Leave-one-year-out	CV00: Leave-one-year-out, new genotypes
XGBoost	G+W+Y+Lon+Lat	Y	Y	0.632	0.522
XGBoost	G+W	Y	N	0.602	0.493
XGBoost	G+Lon+Lat+Y	N	Y	0.658	0.554
LightGBM	G+W+Y+Lon+Lat	Y	Y	0.631	0.521
LightGBM	G+W	Y	N	0.61	0.49
LightGBM	G+Lon+Lat+Y	N	Y	0.663	0.555
Elastic net	G+W+Y+Lon+Lat	Y	Y	0.517	0.453
Elastic net	G+W	Y	N	0.491	0.407
Elastic net	G+Lon+Lat+Y	N	Y	0.564	0.536
LRE model	G+E	N	N	**0.686**	**0.604**
LRE model	G+E+GxE	N	N	0.685	0.602
LRE model	G+S	N	N	0.623	0.562
LRE model	G+S+GS	N	N	0.608	0.56
LRE model	G+Y	N	N	0.487	0.426
LRE model	G+Y+GxY	N	N	0.476	0.429
LRE model	G+E+S+Y+GxS+GxY+GxE	N	N	0.675	0.598
LRE model	G+W	Y	N	0.499	0.431
LRE model	G+E+W	Y	N	0.678	0.59
LRE model	G+W+GxW	Y	N	0.41	0.377
LRE model	G+E+W+GxW	Y	N	0.66	0.556
LRE model	G+E+W+GxW+GxE	Y	N	0.674	0.58
LRE model	G+W	Y	Y	0.513	0.458
LRE model	G+E+W	Y	Y	0.679	0.589
LRE model	G+W+GxW	Y	Y	0.416	0.396
LRE model	G+E+W+GxW	Y	Y	0.661	0.56
LRE model	G+E+W+GxW+GxE	Y	Y	0.676	0.58

Table S9. Weighted average correlation between predicted and observed values across 71 environments for the trait plant height for two cross-validation schemes leaving one site out (CV0-Site, CV00-Site) and for four types of statistical models (XGBoost, LightGBM, Elastic net and linear random effects model, i.e. LRE model) tested with different combinations of predictor variables. The best model for each cross-validation scheme is written in bold.

Type of statistical model	Predictors used	Weather- and soil- based variables included	Longitude and latitude included	CV0: Leave-one-site-out	CV00: Leave-one-site-out, new genotypes
XGBoost	G+W+Y+Lon+Lat	Y	Y	0.700	0.517
XGBoost	G+W	Y	N	0.686	0.512
XGBoost	G+Lon+Lat+Y	N	Y	0.719	0.541
LightGBM	G+W+Y+Lon+Lat	Y	Y	0.704	0.514
LightGBM	G+W	Y	N	0.685	0.515
LightGBM	G+Lon+Lat+Y	N	Y	0.72	0.534
Elastic net	G+W+Y+Lon+Lat	Y	Y	0.576	0.473
Elastic net	G+W	Y	N	0.577	0.447
Elastic net	G+Lon+Lat+Y	N	Y	0.624	0.538
LRE model	G+E	N	N	0.736	0.597
LRE model	G+E+GxE	N	N	0.736	**0.598**
LRE model	G+S	N	N	0.69	0.536
LRE model	G+S+GS	N	N	0.707	0.58
LRE model	G+Y	N	N	0.554	0.38
LRE model	G+Y+GxY	N	N	0.554	0.369
LRE model	G+E+S+Y+GxS+GxY+GxE	N	N	**0.742**	0.59
LRE model	G+W	Y	N	0.591	0.46
LRE model	G+E+W	Y	N	0.732	0.588
LRE model	G+W+GxW	Y	N	0.486	0.363
LRE model	G+E+W+GxW	Y	N	0.717	0.565
LRE model	G+E+W+GxW+GxE	Y	N	0.731	0.579
LRE model	G+W	Y	Y	0.59	0.469
LRE model	G+E+W	Y	Y	0.732	0.588
LRE model	G+W+GxW	Y	Y	0.476	0.384
LRE model	G+E+W+GxW	Y	Y	0.718	0.567
LRE model	G+E+W+GxW+GxE	Y	Y	0.732	0.579

Table S10: Pearson's correlations between predicted and observed values computed within each environment in the CV0-Year prediction problem, using XGBoost with and without environmental data (results ordered by year).

Year_Exp	Pearson's correlation between predicted and observed values - Model XGBoost-G+Lon+Lat+Y	Pearson's correlation between predicted and observed values - Model XGBoost-G+W+Y+Lon+Lat
2014_DEH1	-0.00038	-0.03141
2014_GAH1	0.039908	0.112209
2014_IAH1_early	0.470778	0.412316
2014_IAH1_late	0.244271	0.376256
2014_ILH1	0.326263	0.436469
2014_INH1	0.554022	0.3753
2014_MNH1	-0.14013	0.42382
2014_MOH1	0.35867	0.457647
2014_MOH2	0.389949	0.469562
2014_NEH1	0.175078	0.204411
2014_NEH2	0.227811	0.22328
2014_NYH2	0.494023	0.432915
2014_ONH1	0.270531	0.332591
2014_ONH2	0.526447	0.43561
2014_TXH1	0.468913	0.480222
2014_TXH2	0.275946	0.20704
2014_WIH1	0.548343	0.606907
2015_DEH1	0.428647	0.337348
2015_GAH1	0.100642	0.259561
2015_ILH1	0.292991	0.382949
2015_INH1	0.33941	0.365498
2015_KSH1	0.166681	0.217401
2015_MNH1	0.219478	0.298932
2015_MOH1	0.147063	0.177479
2015_MOH2	0.258198	0.237618
2015_NEH2	0.150283	0.142226
2015_NEH3	0.148109	0.261604
2015_NYH2	0.368203	0.367911
2015_NYH3	0.396606	0.399689
2015_OHH1	0.302886	0.330156
2015_ONH1	0.500552	0.381253
2015_ONH2	0.470438	0.453759
2015_SDH1	0.165344	0.275692
2015_TXH1	0.409079	0.379781
2016_DEH1	0.676664	0.632206
2016_GAH2	0.167941	0.256443

2016_IAH2	0.361859	0.467092
2016_IAH3	0.323082	0.361847
2016_IAH4	0.513045	0.45837
2016_ILH1.a	0.15853	0.20329
2016_ILH1.b	0.113304	0.183547
2016_INH1	0.610272	0.64807
2016_KSH1	0.038255	-0.02406
2016_MIH1	0.630822	0.57595
2016_MNH1	0.499454	0.383406
2016_MOH1	0.519051	0.516576
2016_NEH1	0.559879	0.594455
2016_NEH4	0.336597	0.407543
2016_NYH2	0.17573	0.170988
2016_ONH1	0.512765	0.588222
2016_ONH2	0.488428	0.449989
2016_TXH1	0.488807	0.569038
2016_WIH1	0.656356	0.689881
2016_WIH2	0.627307	0.640923
2017_ARH1	0.080175	0.072308
2017_ARH2	-0.00686	0.028488
2017_COH1	0.384681	0.410604
2017_DEH1	0.759031	0.696925
2017_GAH1	0.235513	0.308306
2017_GAH2	0.406465	0.45164
2017_IAH4	0.659361	0.693042
2017_MIH1	0.734516	0.711509
2017_MOH1	0.589471	0.509746
2017_NYH2	0.430153	0.500422
2017_NYH3	-0.05178	0.157664
2017_ONH1	0.533839	0.523437
2017_TXH1-Dry	0.421615	0.574875
2017_TXH1-Early	0.305317	0.456222
2017_TXH1-Late	0.278046	0.383466
2017_WIH1	0.627729	0.638422
2017_WIH2	0.682127	0.683406

Table S11: Pearson's correlations between predicted and observed values computed within each environment in the CV0-Site prediction problem, using XGBoost with and without environmental data (results ordered by site).

Year_Exp	Pearson's correlation between predicted and observed values - Model XGBoost-G+Lon+Lat+Y	Pearson's correlation between predicted and observed values - Model XGBoost-G+W+Y+Lon+Lat
2017_ARH1	0.083618	0.087805
2017_ARH2	-0.11405	-0.08229
2017_COH1	0.434181	0.395753
2014_NYH2	0.618258	0.645201
2015_NYH2	0.420689	0.46726
2015_NYH3	0.414204	0.504762
2016_NYH2	0.071884	0.13314
2017_NYH2	0.489832	0.510642
2017_NYH3	0.039865	0.194063
2014_ONH2	0.701346	0.680824
2015_ONH2	0.636242	0.626894
2016_ONH2	0.696007	0.488337
2014_ONH1	0.545165	0.498874
2015_ONH1	0.615251	0.466422
2016_ONH1	0.59863	0.583291
2017_ONH1	0.553925	0.59485
2014_IAH1_early	0.492282	0.43794
2014_IAH1_late	0.437323	0.430194
2016_IAH4	0.576048	0.469782
2017_IAH4	0.629009	0.665542
2016_IAH2	0.463735	0.4613
2016_IAH3	0.482565	0.450599
2015_KSH1	0.312603	0.331673
2016_KSH1	0.036547	0.015976
2016_MIH1	0.661474	0.649279
2017_MIH1	0.726516	0.751137
2015_OHH1	0.569874	0.512396
2014_INH1	0.660406	0.479942
2015_INH1	0.410292	0.41781
2016_INH1	0.681134	0.706826
2015_SDH1	0.382275	0.278046
2014_TXH1	0.487586	0.455357
2015_TXH1	0.461609	0.40085
2016_TXH1	0.484127	0.407383
2017_TXH1-Dry	0.551344	0.404471
2017_TXH1-Early	0.415016	0.307181

2017_TXH1-Late	0.270513	0.331329
2014_TXH2	0.50653	0.408112
2014_DEH1	0.000496	-0.03348
2015_DEH1	0.568924	0.647196
2016_DEH1	0.475822	0.576064
2017_DEH1	0.567695	0.675415
2014_GAH1	0.15681	0.158373
2015_GAH1	0.637245	0.587707
2017_GAH1	0.454122	0.182706
2016_GAH2	0.245837	0.167543
2017_GAH2	0.433908	0.475884
2014_ILH1	0.478114	0.551237
2015_ILH1	0.507431	0.540645
2016_ILH1.a	0.191753	0.156859
2016_ILH1.b	0.136002	0.159364
2014_MNH1	0.321004	0.590724
2015_MNH1	0.490254	0.510987
2016_MNH1	0.60069	0.514643
2014_MOH1	0.46074	0.437299
2014_MOH2	0.60346	0.612721
2015_MOH1	0.176224	0.150778
2015_MOH2	0.394699	0.348521
2016_MOH1	0.591315	0.605213
2017_MOH1	0.56974	0.461632
2014_NEH1	0.220867	0.246266
2015_NEH3	0.184895	0.207049
2014_NEH2	0.199181	0.194448
2015_NEH2	0.212923	0.253392
2016_NEH1	0.659393	0.691755
2016_NEH4	0.432744	0.463022
2014_WIH1	0.759711	0.684768
2016_WIH1	0.798492	0.797723
2017_WIH1	0.673311	0.680053
2016_WIH2	0.627422	0.685192
2017_WIH2	0.738874	0.720376
